# The global seroprevalence of anti-*Toxoplasma gondii* antibodies in women who had spontaneous abortion: A systematic review and meta-analysis

**DOI:** 10.1371/journal.pntd.0008103

**Published:** 2020-03-13

**Authors:** Tooran Nayeri, Shahabeddin Sarvi, Mahmood Moosazadeh, Afsaneh Amouei, Zahra Hosseininejad, Ahmad Daryani

**Affiliations:** 1 Toxoplasmosis Research Center, Mazandaran University of Medical Sciences, Sari, Iran; 2 Department of Parasitology, School of Medicine, Mazandaran University of Medical Sciences, Sari, Iran; 3 Student Research Committee, Mazandaran University of Medical Sciences, Sari, Iran; 4 Health Sciences Research Center, Addiction Institute, Mazandaran University of Medical Sciences, Sari, Iran; Istituto Superiore di Sanità, ITALY

## Abstract

**Background:**

*Toxoplasma gondii* (*T*. *gondii*) is an intracellular pathogen that can lead to abortion in pregnant women infected with this parasite. Therefore, the present study aimed to estimate the global seroprevalence of anti-*T*. *gondii* antibodies in women who had spontaneous abortion based on the results of published articles and evaluate the relationship between seroprevalence of anti-*T*. *gondii* antibodies and abortion via a systematical review and meta-analysis.

**Methods:**

Different databases were searched in order to gain access to all studies on the seroprevalence of anti- *T*. *gondii* antibodies in women who had spontaneous abortion and association between seroprevalence of anti-*T*. *gondii* antibodies and abortion published up to April 25^th^, 2019. Odds ratio (OR) and the pooled rate seroprevalence of *T*. *gondii* with a 95% confidence interval (CI) were calculated using the random effects model.

**Results:**

In total, 8 cross-sectional studies conducted on 1275 women who had abortion in present pregnancy, 40 cross-sectional studies performed on 9122 women who had a history of abortion, and 60 articles (involving 35 cross-sectional studies including 4436 women who had spontaneous abortion as case and 10398 as control and 25 case-control studies entailing 4656 cases and 3178 controls) were included for the final analyses. The random-effects estimates of the prevalence of anti-*T*. *gondii* IgG antibody in women who had abortion in present pregnancy and women who had a history of abortion were 33% (95% CI: 17%-49%) and 43% (95% CI: 27%-60%), respectively. In addition, the pooled OR for anti-*T*. *gondii* IgG antibody in cross-sectional and case-control studies among women who had spontaneous abortion were 1.65 (95% CI: 1.31–2.09) and 2.26 (95% CI: 1.56–3.28), respectively. Also, statistical analysis showed that the pooled OR of the risk of anti-*T*. *gondii* IgM antibody 1.39 (95% CI: 0.61–3.15) in cross-sectional and 4.33 (95% CI: 2.42–7.76) in case-control studies.

**Conclusion:**

Based on the results of the current study, *T*. *gondii* infection could be considered a potential risk factor for abortion. It is recommended to carry out further and more comprehensive investigations to determine the effect of *T*. *gondii* infection on abortion to prevent and control toxoplasmosis among pregnant women around the world.

## Author summary

*Toxoplasma gondii* (*T*. *gondii*) is of utmost importance during pregnancy since it can pass through the placental barrier and infect the embryo’s tissues. Consequences of passing the placental barrier and infecting the fetus is abortion, fetal death or severe congenital defects, such as hydrocephaly and chorioretinitis. Although extensive studies have been conducted on the seroprevalence of anti-*T*. *gondii* antibodies in women and its role in abortion, the diversity of studies design, sample size, and the various quality of studies pose daunting challenges to the available information. Therefore, it is essential that this information be updated and synthesized to help physicians and healthcare providers. The results of the current study revealed the high seroprevalence of anti-*T*. *gondii* antibodies in women who had spontaneous abortion and the positive relationship between seroprevalence of anti-*T*. *gondii* antibodies and abortion.

## Introduction

Toxoplasmosis is a serious endemic disease caused by an intracellular parasite called *Toxoplasma gondii* (*T*. *gondii*). According to the seroepidemiological studies, this parasite infects about 15–85% of the total population of the world [1–3]. The only known definitive hosts for *T*. *gondii* are members of family Felidae, including domestic and wild cats. On the other hand, various warm-blooded mammals, including humans and rodents can be the intermediate host of this parasite [4]. Human infections are acquired through several major ways: 1) consumption of undercooked meat especially pork and mutton and unpasteurized milk from infected animals, 2) direct or indirect contact with oocysts from the environment, 3) vertical transmission during pregnancy, 4) blood transfusions, and 5) organ transplants [5–8]. *T*. *gondii* infection is generally asymptomatic in immunologically healthy adults. However, it can cause a variety of life-threatening clinical complications in immunocompromised patients [9]. This parasite is of utmost importance during pregnancy since it can cross the placental barrier to infect embryonic tissues [10, 11]. If this infection occurs during the first and second trimester of pregnancy, it may manifest in severe symptoms, such as low birth weight, hydrocephaly, intracranial calcifications, and retinochoroiditis that are recognizable at birth [12]. On the other hand, infections in the third trimester of pregnancy do generally not show symptoms at birth; however, they may develop intracranial calcifications, hearing impairment, visual disorders, and developmental delay later in life [13]. The global annual incidence rate of congenital toxoplasmosis (CT) is estimated to be 190,100 cases with an approximate incidence rate of 1.5 cases per 1000 live births [14]. Effective factor in transplacental transmission and severity of CT depends on the time of maternal infection [15]. There are two types of miscarriage: sporadic and recurrent [16]. Spontaneous pregnancy loss is a clinical problem of pregnancy occurring in 15% of all clinically recognized pregnancies [17]. The diagnosis of CT for the prevention of abortion is based on laboratory techniques, monitoring the immune response, direct detection of the parasite by animal or tissue inoculation, and molecular techniques [18].

Some of the risk factors of abortion cited in different studies include ethnicity, stress, use of non-steroidal anti-inflammatory or some antidepressant drugs, smoking, use of cocaine, caffeine and alcohol abuse, and obesity [19–29]. Specifically, 15% of early abortions and 66% of late abortions are attributed to infections [30, 31]. *T*. *gondii*, *Toxocara cati*, *Toxocara canis*, *Plasmodium falciparum*, *Escherichia coli*, *Listeria monocytogenes*, *Brucella* species, *Klebsiella pneumonia*, *Rubella*, *Cytomegalovirus*, *Varicella—Zoster Virus*, human immunodeficiency virus, and human papillomavirus are the most common causes of intrauterine infections [32, 33]. Congenital infections, such as CT, are the main causes of miscarriage [33]. Habitual abortion or recurrent miscarriage is the three consecutive pregnancy loss prior to 20 weeks from the last menstrual period [17]. The common causes of habitual abortion include untreated hypothyroidism [34], parental chromosomal abnormalities [35], certain uterine anatomic abnormalities [36], uncontrolled diabetes mellitus [37], immunologic abnormalities [38], infections [39], and environmental factors [40]. The prevalence of *T*. *gondii* infection in pregnant women varies significantly across different continents and countries around the world. Seroprevalence of *T*. *gondii* is reported to be high in Europe, up to 54% in Southern European countries, whereas this value was found to be within 18.5%-92.5% in sub-Saharan Africa [41, 42]. Given the important role of *T*. *gondii* infection in abortion and indefinite rate of *T*. *gondii*-associated abortion around the world, the present study aimed to determine the rate of seroprevalence of anti-*T*. *gondii* antibodies among women who had abortion or a history of abortion, and evaluate the relationship between seroprevalence of anti-*T*. *gondii* antibodies and abortion in women.

## Methods

### Design and protocol registration

A protocol was registered with PROSPERO (No. CRD42019124531) and published and the methods are briefly reported here [43]. We used the preferred reporting items for systematic reviews and meta-analysis guidelines for the performance of this study (S1 Table) [44].

### Search strategy

A literature search was performed using the following electronic databases: PubMed, Scopus, EMBASE, ProQuest, ScienceDirect, Web of Science, and Google Scholar search engine from inception until 25^th^ of April 2019. Search terms are applied alone or in combination as follows: (*Toxoplasma gondii* OR *T*. *gondii* OR toxoplasmosis) AND (abortion OR miscarriage OR fetal loss) AND pregnant women. In addition, the search was restricted to English language articles; therefore, published articles in non-English languages and unpublished studies were not investigated. Moreover, citation lists of relevant papers were checked.

### Inclusion and exclusion criteria

Inclusion criteria entailed English language articles evaluating the effects of *T*. *gondii* on abortion only among human subjects in cross-sectional and case-control studies. On the other hand, we excluded non-original papers (reviews, systematic reviews, editorials or letters), and conference papers. Also, the studies that *T*. *gondii* infection was diagnosed by molecular methods in women who had spontaneous abortion, paraffin-embedded blocks and placenta tissues were excluded from our study.

### Study selection and data extraction

**Fig 1:**
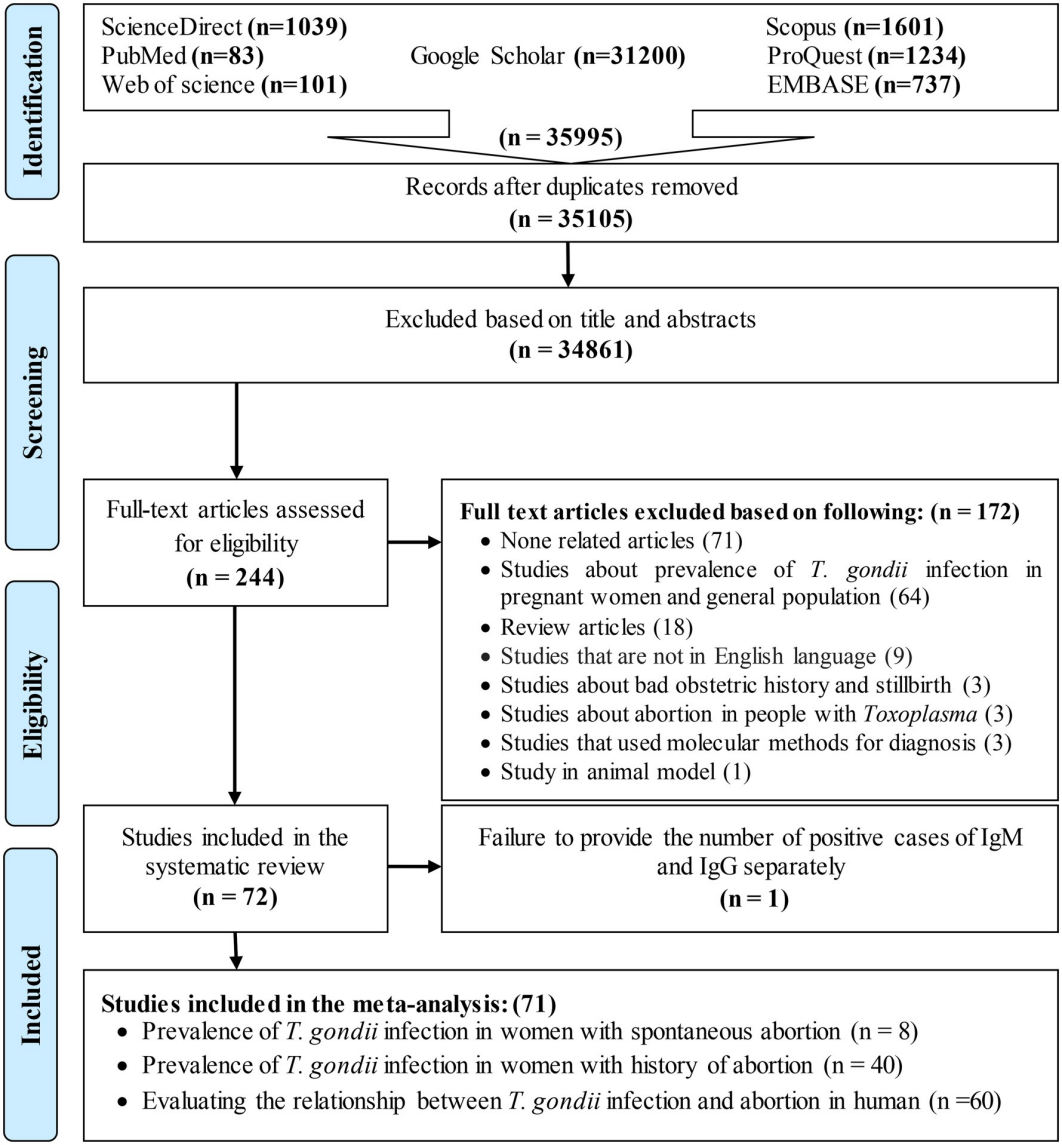
Flow diagram of the study design process.

All retrieved articles were stored in EndNote X9 to organize the summaries. Search results were merged, duplicates were removed automatically and all the titles and abstracts and identified relevant articles were independently scanned by two team members (Fig 1). Two reviewers independently extracted the data using a standardized form. The extracted variables included the name of the first author, year of publication, location of the study, diagnostic method, serological results, number of seropositive cases, as well as the age of the participants.

### Quality assessment

The quality of the articles was assessed independently in terms of selection, comparability, and exposure. Quality was scored using the Newcastle-Ottawa Scales (NOS) [45]. The quality scale was within the range of 0–9 points with a score of ≤ 3 representing a low-quality study. On the other hand, studies with a NOS score > 6 in case-control and > 5 in cross-sectional studies are considered high quality.

### Statistical analysis

The data were analyzed in Stata software (version 14; Stata Corp, College Station, TX, USA). The odds ratio (ORs) was used for this meta-analysis with 95% confidence intervals (CIs) to assess the relationship between seroprevalence of anti-*T*. *gondii* antibodies and abortion in women who had spontaneous abortion. Moreover, the pooled seroprevalence of anti-*T*. *gondii* antibodies was calculated in women with either a recent abortion or a history of abortion. In the forest plots, OR > 1 denotes the positive effect of *T*. *gondii* on abortion, whereas an OR < 1 is indicative of the protective effect of *T*. *gondii* on abortion. In addition, I^2^ statistics were applied to represent the heterogeneity index [46]. Only when I^2^ > 50%, the heterogeneity was considered significant. Publication bias was evaluated through Egger’s test and it was considered significant when P-value was less than 0.05 [47]. Additionally, the sensitivity analysis test was performed by removing the effect of each study on the overall results. In addition, the method of “trim and fill” has been used for a comprehensive evaluation of the possible effects of publication bias. The subgroup analysis and meta-regression test were conducted according to the diagnostic methods and type of study.

## Results

### Study identification and selection

A total of 35105 publications were identified and archived in EndNote (version X9) to organize the resources used in the research process. Duplicates were removed by automation followed by a manual duplicate search. The duplication search included a comparison of the authors’ name, journals name, year of publication, volume number, issue number, page number, and the titles. In the next step, the titles and abstracts of full texts were independently reviewed by two researchers (TN and ZH). Finally, 244 papers were selected for the accurate evaluation of full texts out of which 72 papers were included in the systematic review. One study was not analyzed because it did not accurately quantify the number of IgM and IgG positive cases separately [48], and its data were only systematically presented; therefore, 71 papers were included in the meta-analysis. Eight articles were related to the seroprevalence of anti-*T*. *gondii* antibodies in women who had spontaneous abortion in present pregnancy [49–56]. Moreover, serologic methods were used in six of these studies [49–52, 55, 56]. Two out of 8 articles used both serological and molecular methods in the diagnosis of *T*. *gondii* [53, 54]. The seroprevalence of anti-*T*. *gondii* antibodies in women who had a history of abortion (one or more times) was investigated in 40 articles. In addition, 60 articles were reviewed to estimate the relationship between seroprevalence of anti-*T*. *gondii* antibodies and abortion, out of which 35 articles were cross-sectional studies that examined the seroprevalence of anti-*T*. *gondii* antibodies in women who had a history of abortion; however, it was possible to calculate the ORs for them due to the nature of the studies. Therefore, they were also used to evaluate the relationship between seroprevalence of anti-*T*. *gondii* antibodies and abortion. Fig 1 demonstrates the process of articles screening and selection.

### General characteristics of included studies

General characteristics of included studies are depicted in Tables 1–3. These studies which were published between 1971 and 2019 included 25 case-control and 47 cross-sectional studies. As shown in the Tables 1–3, various studies have used the different diagnostic methods, such as enzyme-linked immunosorbent assay (ELISA), direct agglutination test (DAT), complement fixation test (CFT), latex agglutination test (LAT), indirect haemagglutination (IHA), haemagglutination (HA), indirect immunofluorescence assay (IFA), enzyme immunoassay (EIA), lateral flow immunoassay (LFIA), avidity test, Remington test, mini VIDAS technique, and one step advanced quality. Participants in the majority of articles were ascertained by ELISA (50 studies). Some studies used two or more diagnostic methods for *T*. *gondii* infection. It is noted that most researchers performed the tests using commercial kits and some of them used in house tests.

**Table 1:** Characteristics of the included studies for seroprevalence of anti-*T*. *gondii* antibodies in women who had a history of abortion.

First author	Publication year	Place of study	Type of study	Method (s)	Test	Sample size (n)	IgG+ n (%)	IgM+ n (%)	Age (years)
Kimball, 1971 [55]	1971	USA	Cs	DAT CFT	IgG IgM	941	355 (37.72)	--	--
Stray-Pedersen, 1979 [57]	1979	Norway	Cs	DAT IFA	IgG	2048	279 (13.62)	--	≥ 35
Decavalas, 1990 [58]	1990	Greece	Cs	IFA for IgG Remington for IgM	IgG IgM	126	66 (52.38)	0 (0)	--
Singh, 1998 [59]	1998	United Arab Emirates	Cs	IFA	IgG IgM	1823	547 (30)	3 (0.16)	--
Qublan, 2002 [60]	2002	Amman	Cs	IFA	IgG IgM	104	64 (61.53)	--	15–46
Elnahas, 2003 [5]	2003	Sudan	Cs	ELISA	IgG IgM	129	46 (35.65)	--	--
Nissapatorn, 2003 [61]	2003	Malaysia	Cs	ELISA	IgG IgM	14	10 (71.42)	--	15–44
Chopra, 2004 [62]	2004	India	Cs	ELISA	IgM	118		61 (51.69)	15–45
Ertug, 2005 [63]	2005	Turkey	Cs	ELISA IFA DAT Avidity test	IgG	90	33 (36.7)	--	15–40
Barbosa, 2009 [64]	2009	Brazil	Cs	EIA	IgG IgM	71	46 (64.78)	--	13–40
Nijem, 2009 [65]	2009	Palestine	Cs	ELISA	IgG IgM	76	25 (32.89)	14 (18.42)	16–43
Mousa, 2011 [66]	2011	Libya	Cs	ELISA	IgG IgM	117	55 (47.1)	--	18–44
Drueish, 2011 [67]	2011	Iraq	Cs	ELISA	IgG IgM	122	25 (20.49)	17 (13.93)	15–45
Pavlinová, 2011 [32]	2011	Slovak Republic	Cs	EIA	IgG IgM	221	93 (42.1)	4 (1.8)	31.3 ± 5.6
Jasim, 2011 [68]	2011	Iraq	Cs	ELISA	IgG IgM	162	144 (88.88)	148 (91.35)	15–65
Nissapatorn, 2011 [69]	2011	Thailand	Cs	ELISA	IgG IgM	147	43 (29.25)	--	15–45
Hajsoleimani, 2012 [6]	2012	Iran	Cs	ELISA	IgG IgM	423	159 (37.6)	--	> 30
Malarvizhi, 2012 [70]	2012	India	Cs	ELISA	IgG IgM	67	12 (17.91)	(11.94)	> 40
Padmavathy, 2013 [71]	2013	India	Cs	ELISA	IgG IgM	47	27 (57.44)	4 (8.51)	19.36
Ebrahimzadeh, 2013 [72]	2013	Iran	Cs	ELISA	IgG IgM	71	17 (23.94)	--	14–44
Moura, 2013 [73]	2013	Brasil	Cs	IFA ELISA	IgG IgM	92	59 (64.13)	--	14–45
Babaie, 2013 [74]	2013	Iran	Cs	ELISA	IgG IgM	82	31 (37.80)	--	16–47
Chintapalli, 2013 [75]	2013	India	Cs	ELISA	IgG IgM	20	15 (75)	5 (25)	15–34
Alvarado-Esquivel, 2014 [76]	2014	Mexico	Cs	EIA	IgG IgM	326	22 (6.7)	2 (0.6)	35.57 ± 12.43
Almushait, 2014 [77]	2014	Saudi Arabia	Cs	ELISA	IgG IgM	162	71 (43.82)	12 (7.40)	16–41
Abedi, 2015 [78]	2015	Iran	Cs	ELISA	IgG	300	111 (37)	--	16–39
Awoke, 2015 [42]	2015	Ethiopia	Cs	LAT	IgG IgM	95	29 (30.5)	--	15–44
Gelaye, 2015 [79]	2015	Ethiopia	Cs	LAT	IgG IgM	71	62 (87.3)	--	15–35
Alvarado-Esquivel, 2015 [80]	2015	Mexico	Cs	EIA	IgG IgM	43	2 (4.7)	--	16–50
Anubhuti, 2015 [81]	2015	India	Cs	LFIA	IgG IgM	60	12 (20)	0 (0)	21–35
Mohamed, 2016 [82]	2016	Saudi Arabia	Cs	EIA	IgG IgM	126	23 (18.25)	0 (0)	16–40
Mohaghegh, 2016 [83]	2016	Iran	Cs	ELISA	IgG IgM	35	35 (100)	--	18–45
Imam, 2016 [84]	2016	Egypt	Cs	ELISA	IgG IgM	112	22 (19.6)	--	15–49
Nazir, 2017 [85]	2017	Pakistan	Cs	ELISA	IgG	93	31 (33.33)	--	≥ 36
Yasmeen, 2017 [86]	2017	India	Cs	ELISA	IgG IgM	39	10 (25.6)	--	18–35
Negero, 2017 [41]	2017	Ethiopia	Cs	LAT	IgG IgM	207	176 (83.8)	--	15–34
Matin, 2017 [87]	2017	Iran	Cs	ELISA Nested-PCR	IgG IgM	200	86 (43)	8 (4)	16–41
Costa, 2018 [88]	2018	Brazil	Cs	ELISA	IgG IgM	89	64 (71.9)	--	> 19
Hafez Hassanain [89]	2018	Egypt	Cs	ELISA	IgG IgM IgG avidity	47	20 (42.5)	--	15–44
Rashno, 2019 [90]	2019	Iran	Cs	ELISA PCR	IgG IgM	6	3 (50)	0 (0)	--

Cs: cross-sectional, DAT: direct agglutination test, CFT: complement fixation test, LAT: latex agglutination test, ELISA: enzyme-linked immunosorbent assay, IFA: indirect immunofluorescence assay, EIA: enzyme immunoassay, LFIA: lateral flow immunoassay, PCR: polymerase chain reaction, IgG: immunoglobulin G, IgM: immunoglobulin M

**Table 2:** Characteristics of the included studies for seroprevalence of anti-*T*. *gondii* antibodies in women who had abortion in present pregnancy.

First author	Publication year	Place of study	Type of study	Method (s)	Test	Sample size (n)	IgG+ n (%)	IgM+ n (%)	Age (years)
Kimball, 1971 [55]	1971	USA	Cs	DAT CFT	IgG IgM	260	109 (41.9)	0 (0)	--
Sanghi, 1997 [52]	1997	United Kingdom	Cs	LAT ELISA	IgG IgM	85	0 (0)	0 (0)	--
Hadi, 2011 [56]	2011	Iraq	Cs	Mini VIDAS technique	IgG IgM	190	11 (5.78)	24 (12.63)	15–45
Amin, 2012 [49]	2012	Iran	Cs	ELISA	IgG IgM IgA	264	99 (37.5)	21 (8.0)	14–57
Tammam, 2013 [50]	2013	Egypt	Cs	ELISA	IgG IgM	76	35 (46.1)	14 (18.4)	19–36
Vado-Solis, 2013 [53]	2013	Mexico	Cs	ELISA PCR	IgG IgM	100	32 (32)	2 (2)	25.3 ± 7.3
Hernández-Cortazar, 2016 [54]	2016	Mexico	Cs	ELISA Q-PCR Nested PCR	IgG IgM	161	95 (59)	6 (3.72)	--
El Aal, 2018 [51]	2018	Egypt	Cs	ELISA Q-PCR LAMP	IgG IgM	139	62 (44.6)	--	21–34

Cs: cross-sectional, DAT: direct agglutination test, CFT: complement fixation test, LAT: latex agglutination test, ELISA: enzyme-linked immunosorbent assay, PCR: polymerase chain reaction, Q-PCR: quantitative PCR, LAMP: loop-mediated isothermal amplification, IgG: immunoglobulin G, IgM: immunoglobulin M

**Table 3:** Description of the studies included looking for an association between seroprevalence of anti-*T*. *gondii* antibodies and abortion.

First author	Publication year	Place of study	Type of study	Method (s)	Test	N	Case (n)	Case & IgG+ (n, %)	Case & IgM+ (n, %)	Control (n)	Control & IgG+ (n, %)	Control & IgM+ (n, %)	Age (years)
Kimball, 1971 [55]	1971	USA	Cs	DAT CFT	IgG IgM	4832	941	355 (37.72)	--	3891	1206 (31)	--	P: -- C: 25
Stray-Pedersen, 1977 [91]	1977	Norway	Cc	DAT IFA CFT	IgG	216	157	39 (25)	--	59	11 (19)	--	P: 29.4 C: 29.4
Lolis, 1978 [92]	1978	Greece	Cc	HA IFA	IgG IgM IgA	232	152	62 (40.8)	--	80	10 (12.5)	--	--
Decavalas, 1990 [58]	1990	Greece	Cs	IFA for IgG Remington for IgM	IgG IgM	303	126	66 (52.38)	0 (0)	177	88 (49.71)	0 (0)	P: 27.8 C: 25.9
Galvan Ramirez, 1995 [93]	1995	Mexico	Cc	ELISA	IgG IgM	155	105	48 (44.9)	35 (33.3)	50	13(26.01)	1 (1.9)	P: 32.3 ± 7 C: 31.7 ± 7.1
Sahwi, 1995 [94]	1995	Egypt	Cc	IHA	IgG IgM	140	100	37 (37)	19 (19)	40	4 (10)	3 (7.5)	P: 30 ± 5.975 C: 28 ± 5.236
Djurkovic-Djakovic, 1995 [95]	1995	Yogoslavi	Cc	DAT	IgG IgM	2315	1747	570 (32.62)	94 (5.38)	568	193 (33.97)	27 (4.75)	14–45
Al Hamdani, 1997 [96]	1997	Iraq	Cc	IHA IFA	IgG IgM	200	81	15 (18.5)	--	119	7 (5.9)	--	P: 29 ± 7.9 C: 27 ±6.93
Zargar, 1998 [97]	1998	India	Cc	ELISA	IgM	454	285	--	141 (49.47)	169	--	15 (8.87)	P: 27.73 ± 4.57 C: 28.59 ±5.13
Qublan, 2002 [60]	2002	Amman	Cs	IFA	IgG IgM	280	104	64 (61.53)	--	176	68 (38.63)	--	15–46
Elnahas, 2003 [5]	2003	Sudan	Cs	ELISA	IgG IgM	487	129	46 (35.65)	--	358	120 (33.51)	--	--
Nissapatorn, 2003 [61]	2003	Malaysia	Cs	ELISA	IgG IgM	200	14	10 (71.42)		186	88 (47.3)		15–44
Chopra, 2004 [62]	2004	India	Cs	ELISA	IgM	218	118		61 (51.69)	100		0 (0)	15–45
Nimri, 2004 [98]	2004	France	Cc	ELISA Nested PCR	IgG IgM	248	148	80 (54.0)	4 (2.7)	100	12 (12.0)	0 (0)	P: 28 C: 28
Ertug, 2005 [63]	2005	Turkey	Cs	ELISA IFA DAT Avidity Test	IgG	357	90	33 (36.7)	--	267	78 (29.2)	--	15–40
Surpam, 2006 [99]	2006	India	Cc	ELISA	IgM	119	44	--	12 (27.27)	75	--	1 (1.33)	20–38
Sebastian, 2008 [100]	2008	India	Cc	ELISA	IgG IgM	101	71		36 (50.7)	30	--	6 (20)	15–34
Al-Saeed, 2008 [101]	2008	Iraq	Cc	LAT	IgG IgM	140	120	50 (41.7)	--	20	0 (0)	--	--
Barbosa, 2009 [64]	2009	Brazil	Cs	EIA	IgG IgM	190	71	46 (64.78)	--	119	81 (68.06)	--	13–40
Nijem, 2009 [65]	2009	Palestine	Cs	ELISA	IgG IgM	204	76	25 (32.89)	14 (18.42)	128	32 (25)	22 (17.18)	16–43
AL–Taie, 2010 [102]	2009	Iraq	Cc	ELISA	IgM	88	38	--	15 (39.4)	50	--	6 (12)	> 41
Hassan, 2009 [103]	2009	Iraq	Cc	ELISA	IgG IgM	119	96	7 (7.29)	20 (20.83)	23	2 (8.7)	0 (0)	23.9–28.5
Drueish, 2011 [67]	2011	Iraq	Cs	ELISA	IgG IgM	177	122	25 (20.49)	17 (13.93)	50	12(24)	0 (0)	15–45
Pavlinová, 2011 [32]	2011	Slovak Republic	Cs	EIA	IgG IgM	537	221	93 (42.1)	4 (1.8)	179	45 (25.1)	5 (2.8)	P: 31.3 ± 5.6 C: 29.4 ± 5.6
Jasim, 2011 [68]	2011	Iraq	Cs	ELISA	IgG IgM	300	162	144 (88.88)	148 (91.35)	138	123 (89.13)	122 (88.40)	15–65
Mousa, 2011 [66]	2011	Libya	Cs	ELISA	IgG IgM	143	117	55 (47.1)	--	26	9 (34.6)	--	18–44
Nissapatorn, 2011 [69]	2011	Thailand	Cs	ELISA	IgG IgM	640	147	43 (29.25)	--	493	138 (28)	--	15–45
Hajsoleimani, 2012 [6]	2012	Iran	Cs	ELISA	IgG IgM	500	423	159 (37.6)	--	77	30 (39)	--	> 30
Malarvizhi, 2012 [70]	2012	India	Cs	ELISA	IgG IgM	232	67	12 (17.91)	8 (11.94)	165	11 (4.74)	1 (0.43)	> 40
Elamin, 2012 [104]	2012	Saudi Arabia	Cc	ELISA PCR	IgG IgM	188	94	33 (35.1)	5 (15.2)	94	37 (39.4)	6 (16.2)	--
Ebrahimzadeh, 2013 [72]	2013	Iran	Cs	ELISA	IgG IgM	221	71	17 (23.94)	--	150	51 (34)	--	14–44
Babaie, 2013 [74]	2013	Iran	Cs	ELISA	IgG IgM	419	82	31 (37.80)	--	337	113 (33.5)	--	16–47
Moura, 2013 [73]	2013	Brazil	Cs	IFA ELISA	IgG IgM	400	92	59 (64.13)	--	308	175 (56.81)	--	14–45
Chintapalli, 2013 [75]	2013	India	Cs	ELISA	IgG IgM	32	20	15 (75)	5 (25)	12	1 (8.33)	1 (8.33)	15–34
Hussan, 2013 [105]	2013	Iraq	Cc	ELISA	IgG IgM	255	210	46 (22)	32 (15.23)	45	3 (6.66)	0 (0)	--
Abou-Gabal, 2013 [48]	2013	Egypt	Cc	One step advanced quality	IgG IgM	80	40	--	--	40	--	--	P: 33.13 ± 10.341 C: 33.05 ± 8.941
Siddiqui, 2014 [106]	2014	India	Cc	ELISA	IgG IgM	63	48	--	17 (35.4)	15	--	0 (0)	> 30
Almushait, 2014 [77]	2014	Saudi Arabia	Cs	ELISA	IgG IgM	487	162	71 (43.82)	12 (7.40)	325	118 (36.3)	18 (5.5)	16–41
Sultana, 2014 [107]	2014	Bangladesh	Cc	ELISA	IgG IgM	91	46	8 (17.4)	7 (15.2)	45	4 (8.9)	0 (0)	P: 24.43 ± 4.17 C: 24.56 ±4.36
Abbas, 2014 [108]	2014	Iraq	Cc	ELISA	IgG IgM	130	100	42 (42)	12 (12)	30	0 (0)	0 (0)	--
Awoke, 2015 [42]	2015	Ethiopia	Cs	LAT	IgG IgM	384	95	29 (30.5)	--	289	42 (14.5)	--	15–44
Gelaye, 2015 [79]	2015	Ethiopia	Cs	LAT	IgG IgM	288	71	62 (87.3)	--	217	184 (84.8)	--	15–35
Saki, 2015 [109]	2015	Iran	Cc	ELISA	IgG IgM	260	130	32 (24.6)	1 (0.76)	130	28 (21.5)	0 (0)	--
Kamal, 2015 [110]	2015	Egypt	Cc	ELISA	IgG IgM	149	29	18 (62.0)	--	120	8 (6.66)	--	18–40
Anubhuti, 2015 [81]	2015	India	Cs	LFIA	IgG IgM	120	60	12 (20)	0 (0)	60	3 (5)	0 (0)	21–35
Alvarado-Esquivel, 2015 [80]	2015	Mexico	Cs	EIA	IgG IgM	150	43	2 (4.7)	--	107	12 (11.2)	--	16–50
Ghasemi, 2016 [111]	2016	Iran	Cc	ELISA PCR	IgG IgM	192	82	22 (26.8)	3 (3.6)	110	29 (26.4)	1 (0.9)	P: 28.29 C: 28.58
Mohaghegh, 2016 [83]	2016	Iran	Cs	ELISA	IgG IgM	350	35	35 (100)	--	315	75 (23.8)	--	18–45
Mohamed, 2016 [82]	2016	Saudi Arabia	Cs	ELISA	IgG IgM	326	126	23 (18.25)	0 (0)	200	46 (23)	4 (2)	16–40
Nazir, 2017 [85]	2016	Pakistan	Cs	ELISA	IgG	403	93	31 (33.33)	--	310	40 (12.90)	--	≥ 36
Imam, 2016 [84]	2016	Egypt	Cs	ELISA	IgG IgM	138	112	22 (19.6)	--	26	4 (15.4)	--	15–49
Rasti, 2016 [112]	2016	Iran	Cc	ELISA	IgG IgM	179	81	22 (27.2)	1 (1.2)	98	28 (28.6)	2 (2)	P: 28.2 C: 28.6
Yasmeen, 2017 [86]	2017	India	Cs	ELISA	IgG IgM	251	39	10 (25.6)	--	212	43 (20.3)	--	18–35
Negero, 2017 [41]	2017	Ethiopia	Cs	LAT	IgG IgM	369	207	176 (83.8)	--	162	34 (16.2)	--	15–34
Matin, 2017 [87]	2017	Iran	Cs	ELISA Nested-PCR	IgG IgM	200	58	31 (53.44)	0 (0)	142	55 (38.73)	8 (5.63)	16–41
Costa, 2018 [88]	2018	Brazil	Cs	ELISA	IgG IgM	352	89	64 (71.9)	--	263	189 (71.8)	--	> 19
Çakmak, 2018 [113]	2018	Turkey	Cc	ELISA	IgG IgM	1240	412	125 (30.6)	27 (6.6)	828	157 (19.2)	35 (4.2)	P: 27.6 ± 11.4 C: 29.1 ± 9.87
Hafez Hassanain, 2018 [89]	2018	Egypt	Cs	ELISA	IgG IgM IgG avidity	388	47	20 (42.5)	--	341	59 (17.3)	--	15–44
Rashno, 2019 [90]	2019	Iran	Cs	ELISA PCR	IgG IgM	98	6	3 (50)	0 (0)	92	31 (33.69)	0 (0)	--
Kheirandish, 2019 [114]	2019	Iran	Cc	ELISA	IgG IgM	480	240	114 (47.5)	8 (3.3)	240	111 (46.3)	1 (0.4)	P: 27 ± 6.499 C: 27.01 ± 6.459

Cs: cross-sectional, CC: case-control, DAT: direct agglutination test, CFT: complement fixation test, LAT: latex agglutination test, ELISA: enzyme-linked immunosorbent assay, IHA: indirect haemagglutination, HA: haemagglutination, IFA: indirect immunofluorescence assay, EIA: enzyme immunoassay, LFIA: lateral flow immunoassay, PCR: polymerase chain reaction, IgG: immunoglobulin G, IgM: immunoglobulin M, Case: women who had abortion, Control: women who had not abortion

In addition, the included articles in the present meta-analysis showed an acceptable quality (i.e., ≥ 3 for cross-sectional studies and ≥ 4 for case-control studies). S2 Table represents the quality score of different eligible studies.

### Seroprevalence of anti-*T*. *gondii* antibodies in women who had a history of abortion

**Fig 2:**
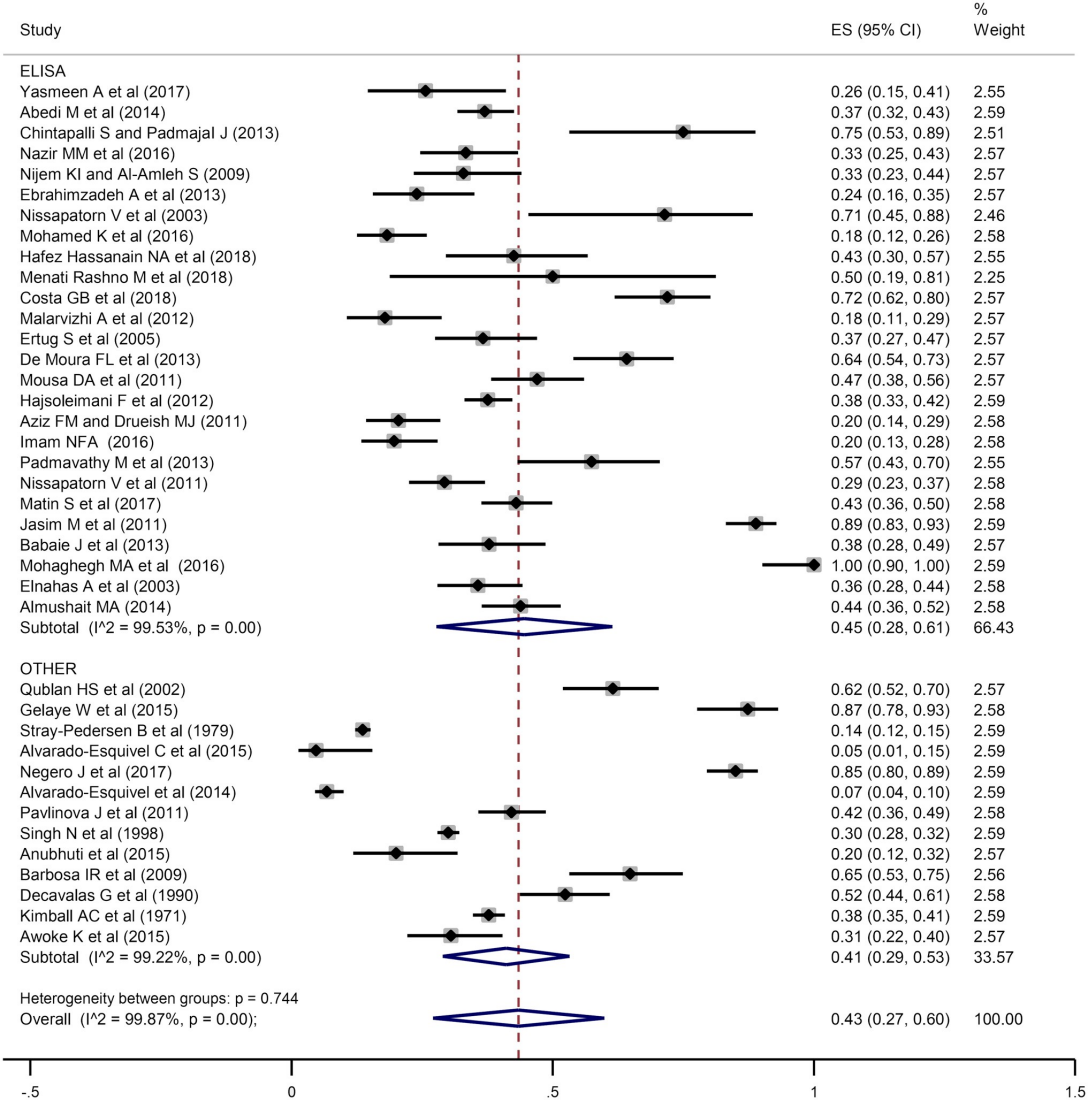
The reported seroprevalence of anti- *T*. *gondii* IgG antibody in women who had a history of abortion. The horizontal lines define the reported 95% confidence interval for the seroprevalence in each study, and the diamond below the graph shows the pooled seroprevalence.

A total of 9004 women who had a history of abortion were investigated for the seroprevalence of anti*-T*. *gondii* IgG antibody in 39 articles, out of which 2930 were positive using several serological methods. In addition, 16 studies entailing 3662 women who had spontaneous abortion were reviewed for the seroprevalence of anti-*T*. *gondii* IgM antibody out of which 286 women were seropositive. According to forest plot diagram in Fig 2 and S1 Fig, the pooled seroprevalence of anti- *T*. *gondii* IgG and IgM antibodies in women who had a history of abortion based on a random effects model was estimated at 43% (95% CI: 27%-60%) and 3% (95% CI: 3%-4%), respectively. Furthermore, I^2^ statistic revealed a significant heterogeneity among the studies (I^2^ = 99.87%, P = 0.00). Moreover, subgroup analysis results based on diagnostic methods showed that heterogeneity for ELISA and other methods (DAT, CFT, LAT, IFA, EIA, LFIA, and Remington test) were I^2^ = 99.53%, P = 0.00 and I^2^ = 99.22%, P = 0.00, respectively. In addition, heterogeneity between groups was P = 0.744 (Fig 2).

### Seroprevalence of anti*-T*. *gondii* antibodies in women who had abortion in present pregnancy

**Fig 3:**
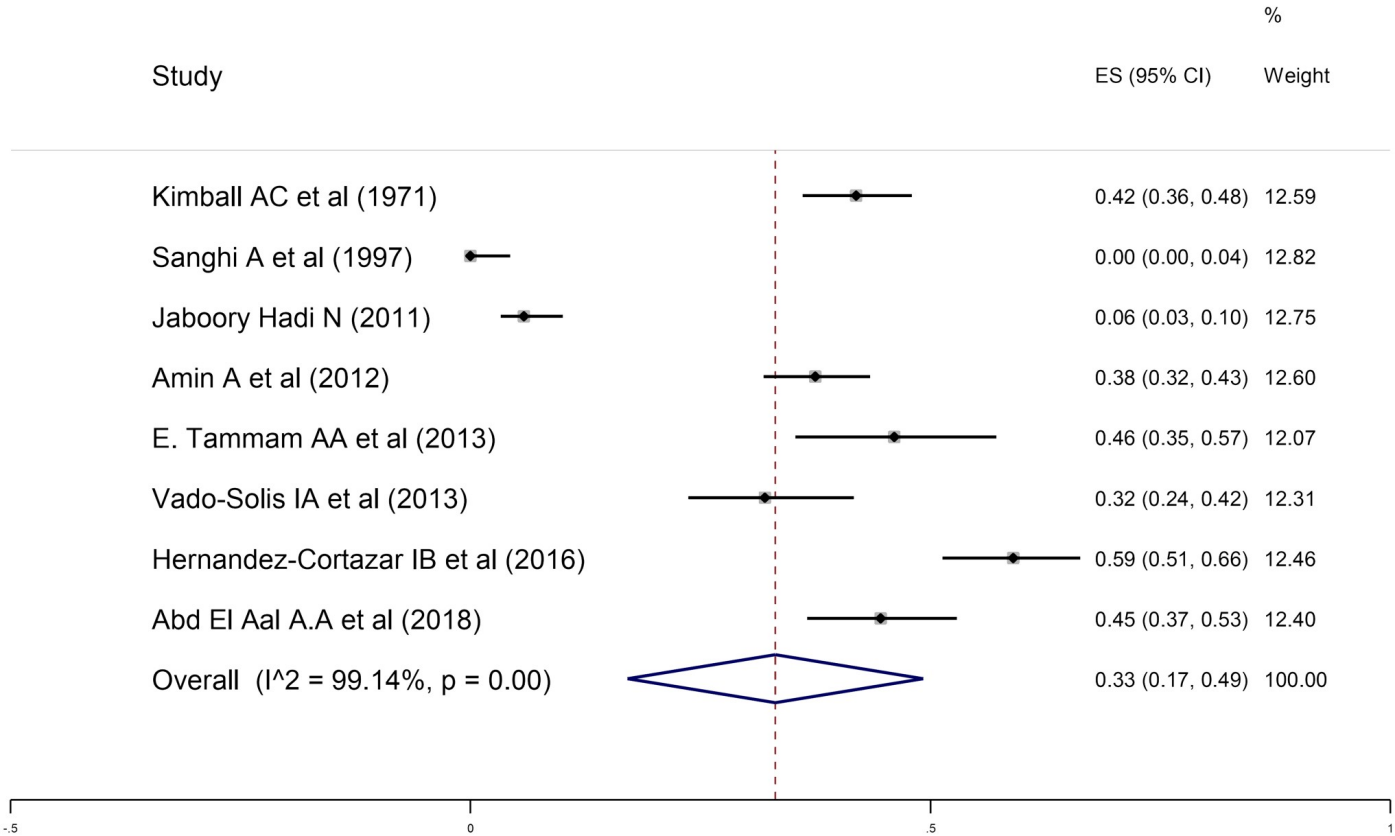
The reported seroprevalence of anti- *T*. *gondii* IgG antibody in women who had abortion in present pregnancy.

A total number of 1275 women who had spontaneous abortion in present pregnancy were examined for the seroprevalence of anti- *T*. *gondii* antibodies using different serologic tests. 1275 serum samples in eight studies and 1136 serum samples of women who had abortion in present pregnancy in seven studies were evaluated for anti- *T*. *gondii* IgG and IgM antibodies using serologic tests out of which 443 and 67 cases were positive for anti- *T*. *gondii* IgG and IgM antibodies, respectively. The pooled seroprevalence rates of anti- *T*. *gondii* IgG and IgM antibodies in women who had abortion in present pregnancy using a random-effects model were determined to be 33% (95% CI: 17%-49%) and 1% (95% CI: 1%-2%), respectively (Fig 3 and S2 Fig). The results of heterogeneity test in different studies for IgG and IgM antibodies were I^2^ = 99.14%, P = 0.00; I^2^ = 92.06%, P = 0.00, respectively.

### Relationship between anti-*T*. *gondii* IgG antibody and abortion

**Fig 4:**
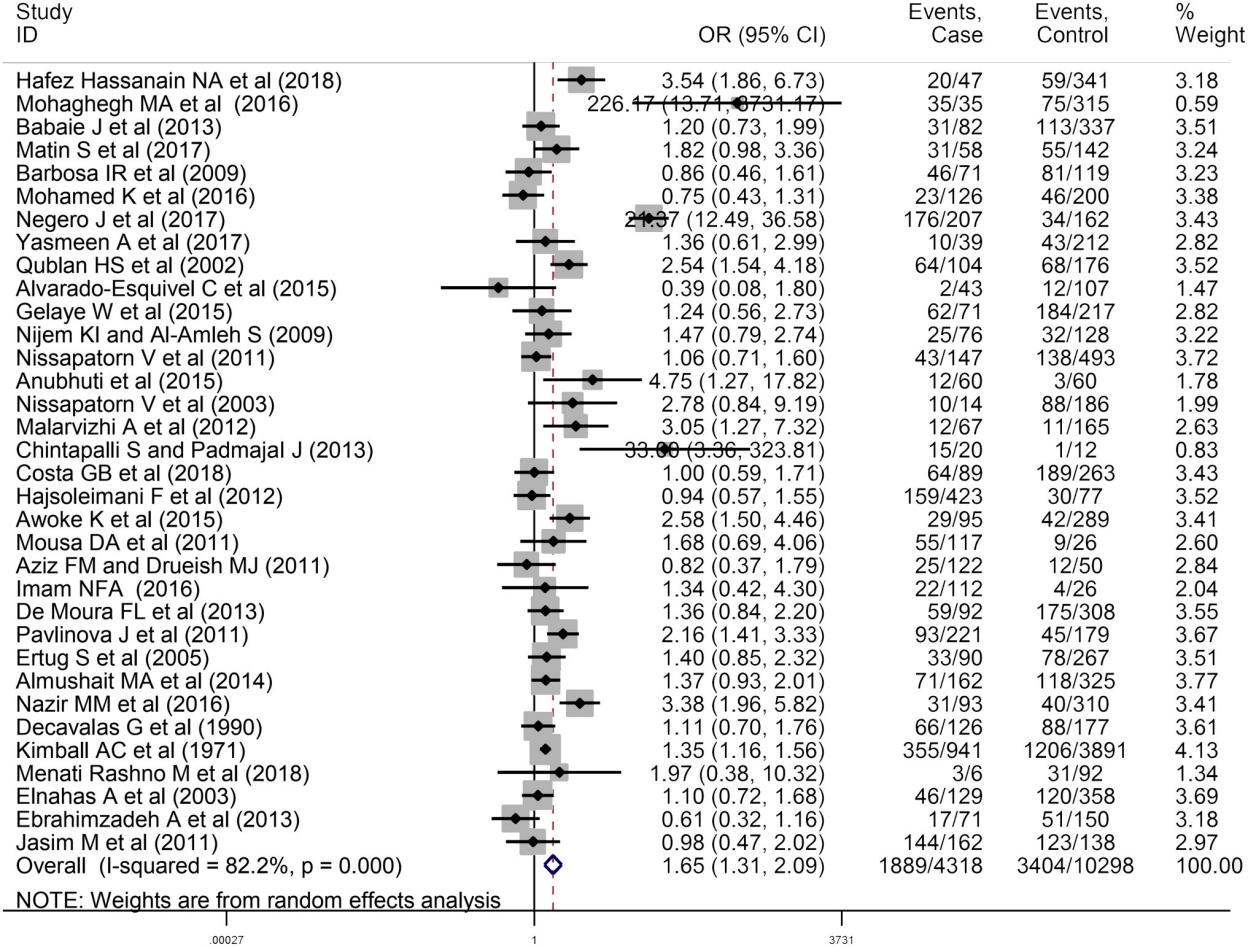
Forest plot diagram of cross-sectional studies showing IgG seropositivity rates of *T*. *gondii*.

**Fig 5:**
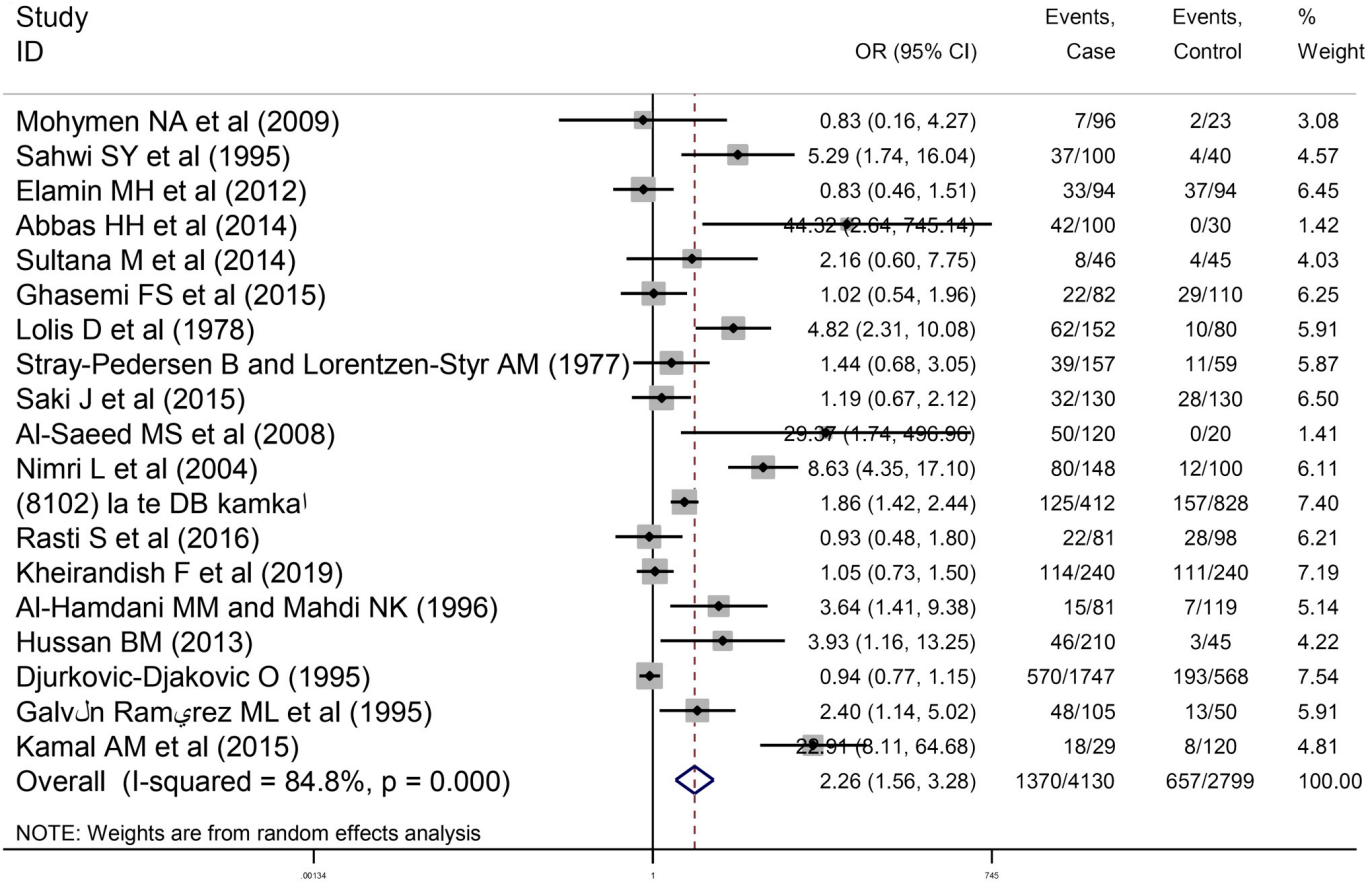
Forest plot diagram of case-control studies showing IgG seropositivity rates of *T*. *gondii*.

A total of 53 studies entailing 8448 women who had spontaneous abortion and 13097 control individuals were evaluated in the current study. This meta-analysis included 34 cross-sectional studies evaluating anti-*T*. *gondii* IgG antibody and abortion with 4318 women who had spontaneous abortion (1889 positive for anti-*T*. *gondii* antibodies) and 10298 women as the control group (3404 positive for anti-*T*. *gondii* antibodies). Moreover, 19 case-control articles evaluating anti-*T*. *gondii* IgG antibody and abortion were entered into the meta-analysis including 4130 women who had spontaneous abortion (1370 positive for anti- *T*. *gondii* antibodies) and 2799 controls (657 positive for anti- *T*. *gondii* antibodies). As depicted in the forest plot diagram, the pooled ORs of the risk of anti-*T*. *gondii* IgG antibody investigated in women who had spontaneous abortion in cross-sectional and case-control studies were 1.65 (95% CI: 1.31–2.09) and 2.26 (95% CI: 1.56–3.28), respectively (Figs 4 and 5). Additionally, the test of heterogeneity revealed significant heterogeneity among cross-sectional and case-control studies as I^2^ = 82.2%, P = 0.00 and I^2^ = 84.8%, P = 0.00, respectively. Publication bias was assessed using Egger’s test in cross-sectional studies (P = 0.194) and no publication bias was observed (S3 Fig). On the other hand, the results of Eggert’s test were statistically significant in case-control studies (P = 0.007) (S4 Fig). In addition, there was no change in the results of pooled random-effects analysis corrected by using “trim and fill” method (S5 Fig). The robustness and reliability of the results of this meta-analysis were indicated by sensitivity analysis (S6 and S7 Figs). In order to improve the interpretation of the meta-analysis results, a meta-regression test was performed based on the type of study revealing the significant effect of type of study on the heterogeneity of the studies (P = 0.032).

### Relationship between anti-*T*. *gondii* IgM antibody and abortion

**Fig 6:**
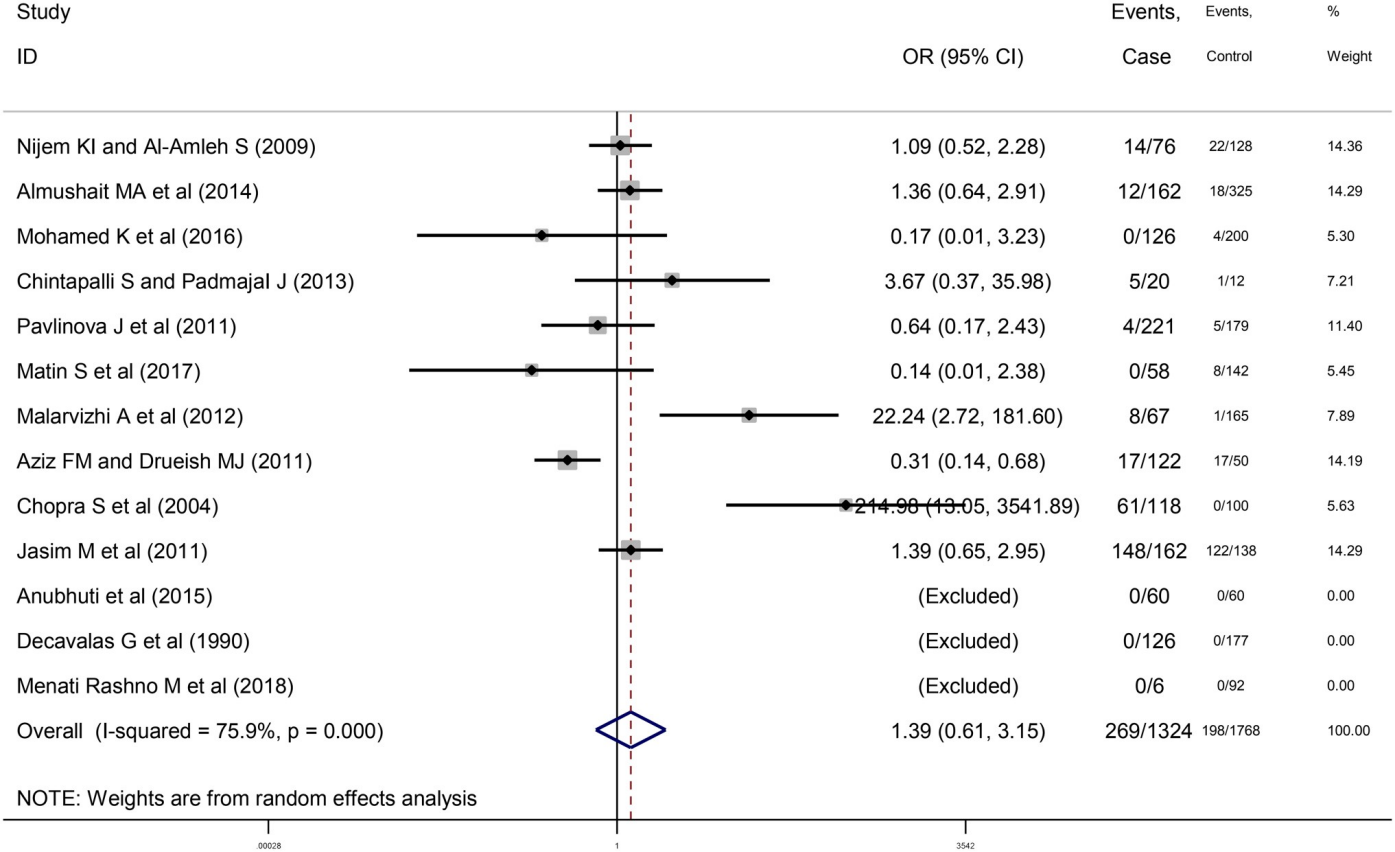
Forest plot diagram of cross-sectional studies showing IgM seropositivity rates of *T*. *gondii*.

**Fig 7:**
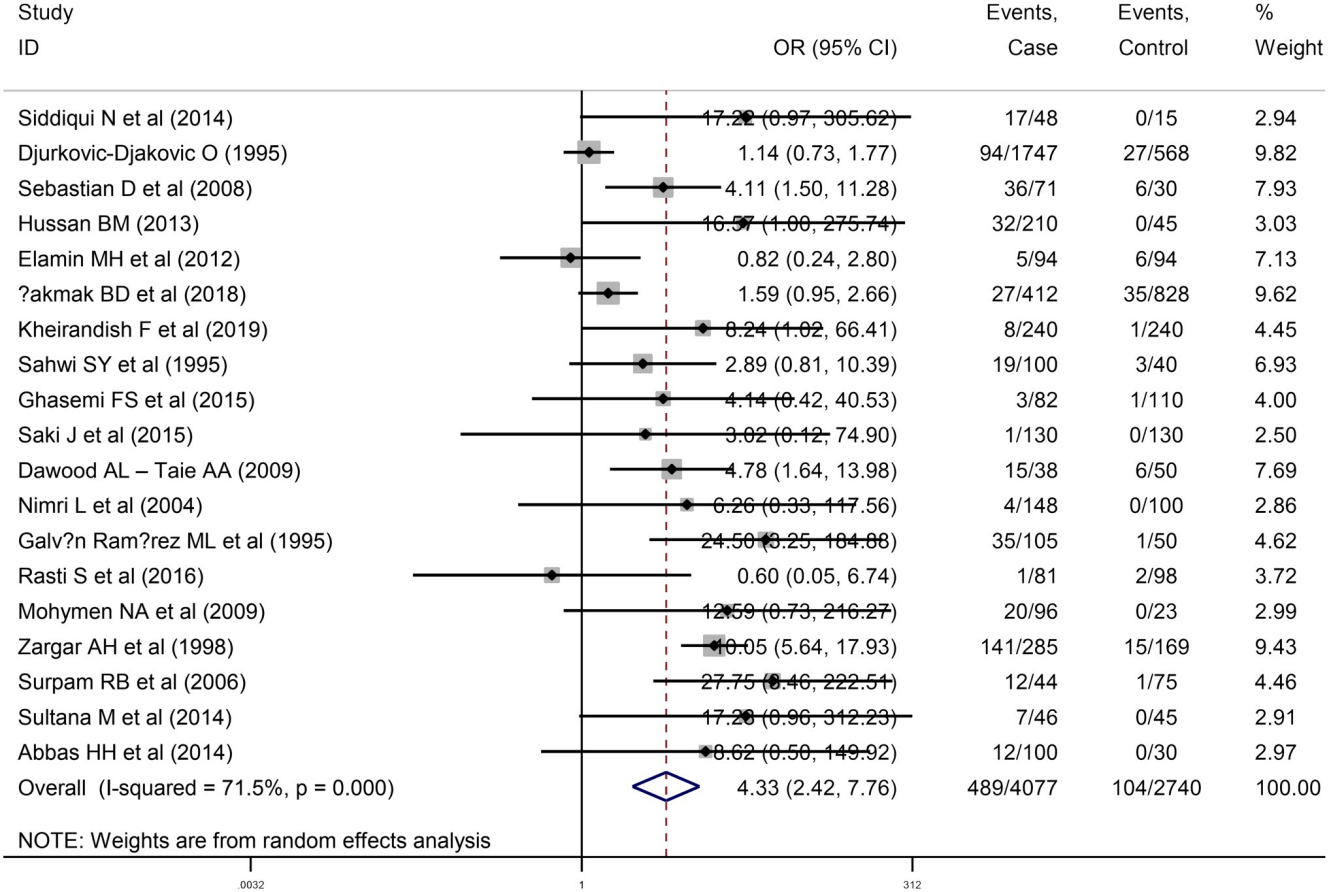
Forest plot diagram of case-control studies showing IgM seropositivity rates of *T*. *gondii*.

A total of 29 papers were entered into the meta-analysis including 1132 women who had spontaneous abortion (269 positive for anti- *T*. *gondii* antibodies) and 1439 controls (198 positive for anti- *T*. *gondii* antibodies) in 10 cross-sectional studies, as well as 4077 women who had spontaneous abortion (489 positive for anti- *T*. *gondii* antibodies) and 2740 controls (104 positive for anti- *T*. *gondii* antibodies) in 19 case-control studies evaluating anti-*T*. *gondii* IgM antibody. The results of the meta-analysis indicated a common OR of the risk of anti-*T*. *gondii* IgM antibody 1.39 (95% CI: 0.61-3.15) in cross-sectional studies (Fig 6) and 4.33 (95% CI: 2.42–7.76) in case-control ones (Fig 7). Moreover, the results illustrated a significant heterogeneity in cross-sectional and case-control studies (I^2^ = 75.9%, P = 0.00 and I^2^ = 71.5%, P = 0.00), respectively.

The results of Egger’s test were not statistically significant in cross-sectional studies (P = 0.36), which indicates no publication bias. However, the funnel plot shows asymmetric pattern (S8 Fig). Based on the results of “trim and fill” method, publication bias does not appear to have a significant impact on the results (S9 Fig). On the other hand, the results of Egger’s test were statistically significant in case-control studies (P = 0.02) (S10 Fig). According to the results of “trim and fill” method, publication bias does not have a significant effect on the results of the pooled random-effects analysis (S11 Fig). The results confirmed the reliability and stability of the present meta-analysis after the performance of sensitivity analyses (S12 and S13 Figs). In addition, we performed the meta-regression analysis based on the type of study suggesting type of study had no significant effect on the heterogeneity among the studies included in the present meta-analysis (P = 0.77).

## Discussion

Fetuses are at high risk of this parasite during acute infection due to *Toxoplasma*'s ability to cross the placental barrier and infect the fetus before it can acquire immunity [115]. This systematic review and meta-analysis investigated the seroprevalence of anti- *T*. *gondii* antibodies in women who had abortion in present pregnancy or a history of abortion and the relationship between the seroprevalence of anti- *T*. *gondii* antibodies and abortion. The obtained results indicated that the overall estimation for the prevalence of anti- *T*. *gondii* IgG antibodies in women who had a history of abortion (43%; 95% CI: 27%-60%) was higher than those with present abortion (33%; 95% CI: 17%-49%). According to Figs 2 and 3, the highest and lowest seroprevalence rates of anti- *T*. *gondii* antibodies in women who had a history of abortion were related to the studies performed by Mohaghegh *et al*. as 100% (95% CI: 0–100%) [83] and Alvarado-Esquivel *et al*. as 5% (95% CI: 1–15%) [80]. Additionally, the highest and lowest seroprevalence rates of anti- *T*. *gondii* antibodies in women who had abortion in present pregnancy were observed in studies conducted by Hernández-Cortazar *et al*. as 59% (95% CI: 51–66%) [54] and Sanghi *et al*. as 0% (95% CI: 0–4%) [52]. IgG seropevalence is dependent on age and the risk of fetal loss increases after the age of 35 years. The risk of fetal loss is 9% in women aged 20–24 years and increases to 75% among women aged 45 and over. Because in the included articles the relationship of age and the seropositivity of *T*.*gondii* antibodies was not evaluated, we avoided the analysis of this main risk factor and this can be considered as a basic gap.

The prevalence of this infection varies according to geographical area and differences in climate, dietary habits, hygiene, and susceptibility of the host [116]. The global prevalence of *T*. *gondii* infection in pregnant women is within the range of 7%-51.3% and seroprevalence in women with abnormal pregnancies is within the range of 17.5–52.3% [93, 97]. The overall prevalence of *T*. *gondii* varies from country to country. Moreover, in some countries it has declined dramatically over the years. For example, in France, the prevalence of *T*. *gondii* infection in pregnant women in the 1960s was 84%, in 1995, it was 54% and in 2003, it was 44%. Since France operates a congenital toxoplasmosis surveillance system; so, this system appears to be an essential tool for evaluating the efficacy of new screening strategies [117].

Moreover, this study assessed the relationship between seroprevalence of anti- *T*. *gondii* antibodies and abortion. The results revealed that the ORs of prevalence of anti-*T*. *gondii* IgG antibodies in women who had spontaneous abortion were 1.65 (95% CI: 1.31–2.09) in cross-sectional and 2.26 (95% CI: 1.56–3.28) in case-control studies, compared to control group. In all of these analyses, heterogeneity was significant (I^2^ > 50%). Variation in these studies in terms of inclusion and exclusion criteria, the diverse populations, the difference in the methods of selection of the target populations, the age of the participants in the studies, the type of study (cross-sectional or case-control studies) may contribute to heterogeneity among studies. Therefore, a meta-regression test was performed to investigate the impact of the type of study on heterogeneity. The results suggested that the type of study had a significant effect on the assessment of the relationship between anti-*T*. *gondii* IgG antibody and abortion. However, this effect was not significant for anti-*T*. *gondii* IgM antibody. Based on results of the Egger’s test, there is no significant publication bias in cross-sectional studies. However, the results of Eggert’s test were statistically significant in case-control studies. These findings are likely to be related to the variability of the results of preliminary studies entered the meta-analysis. It is worthy to note that usually language-bias can be a possible reason for publication bias since only English-language articles were used in this study. As the results of the "trim and fill" method showed that the publication bias did not have a significant effect on the study results; so, the results presented in this paper seem to be valid and it is not necessary to use other languages. Furthermore, English abstracts of articles with non- English languages, due to the lack of sufficient information for analysis, were not included in our study.

Out of 72 studies, 43 were conducted in Asia, 12 in Africa, 9 in America and 8 in Europe and none in Australia and many European and American countries have no published articles in this field. There are several risk factors for the prevalence of *T*. *gondii* in women who had spontaneous abortion, such as ethnicity, socioeconomic status, history of contact with cats, raw meat consumption, strain’s virulence of *T*. *gondii*, immunological competence of the mother, smoking, and alcohol abuse. However, these risk factors were not specifically investigated in most studies and it was not possible to perform meta-analysis these risk factors.

It is important to determine the timing of abortion in relation to the measurement of seroprevalence of anti- *T*. *gondii* antibodies; because, occurrence of infection in the first trimester, when hormone levels are low and there is little helper T cell type 2 (Th2) bias, reduces the chance of transmission to the fetus but increases the likelihood of miscarriage. In contrast, the occurrence of infection in the third trimester, when there is a strong Th2 bias, makes abortion unlikely, but increases congenital transmission. The helper T cell type 1 responses caused by *T*. *gondii* infection in early pregnancy may induce miscarriage. In contrast, the strong Th2 bias and decreased natural killer cells, macrophages and CD8+ T cells function occur in the late stages of pregnancy that may contribute to parasite survival and increase the chance of congenital transmission [118]. However, due to the lack of evaluation of this risk factor in most studies assessing the relationship between abortion and the seroprevalence of anti- *T*. *gondii* antibodies, or incomplete data in some studies, the timing of abortion was not analyzed and this is considered as a major gap. On the other hand, in cross-sectional studies, it is not known when the abortion or the seroconversion happened.

Results of IgG calculated in this study (43% and 33%) do not provide sufficient data for the evaluation of CT. This can be due to the fact that IgG appears approximately 2 weeks after IgM, reaches peak levels within 6–8 weeks, and starts to slowly decrease to lower levels after 1 year until the end of infected subject’s life due to the persistence of latent cysts in immune-privileged organs [18, 119, 120].

In the current study, the overall prevalence of anti- *T*. *gondii* IgM antibodies in women who had abortion in present pregnancy or a history of abortion was estimated at 1% (95% CI: 1%-2%) and 3% (95% CI: 3%-4%), respectively. IgM antibody is an indicator of recent infection and detection of specific IgM antibody can assist in the determination of acute infection. It is usually detectable 1 week after infection and declines more rapidly, compared to IgG antibody. Level of IgM antibody increases to reach peak levels after 1–3 months. There is a slow decline in antibody levels over the next 9 months until negativation [121, 122]. It was revealed that IgM antibodies can be detected for 12 years following the acute phase of infection [121]. On the other hand, autoimmune antibodies, such as rheumatoid factor and antinuclear antibodies, non-specific in vitro binding and acute viral infection, can cause false-positive IgM results up to 60% and results of commercial kits used in reference laboratories. This can be viewed as the limitation of this method since it cannot be determined whether the patient has contracted this infection a few months ago or is in the acute phase of the disease which can put the fetus at risk [123, 124].

Positive IgM test results should be confirmed in reference laboratories using specific tests, such as IgG avidity, or by evaluation of IgA and IgE antibodies [125]. When the IgM serologic reaction is positive in an asymptomatic patient, the IgG avidity test is an auxiliary test to distinguish between acute and chronic infection. Avidity antibodies are low in pregnant women who were infected at least 3–5 months earlier. Low-avidity may persist for 1 year. In these cases, the interpretation of the results needs more precision [125, 126].

It is better to study IgA antibody in pregnant women to continue the evaluation of the acute form of *T*.*gondii* infection. IgA production is similar to that of IgM and it appears during the first week, whereas IgA antibody peak occurs later than IgM and persist more than 3 or 4 months following primary infection; therefore, they disappear earlier than IgM [127]. IgA may persist for more than a year [18, 120, 127]. Therefore, it is not also sufficient in the detection of acute infection in adults [128].

On the other hand, specific IgE antibodies are produced rapidly and remain detectable less than 4 months after infection by ELISA in sera of adults with acute infection, neonates infected with congenital infection, and children with chorioretinitis [129]. IgE antibody may be helpful in the diagnosis of lymphadenopathies, and the persistence of IgE can be an indicator of active toxoplasmosis [130]. Although IgE seropositivity occurs for a briefer period than IgM or IgA antibodies and it is useful for the diagnosis of the acute form [131, 132], it does not have enough sensitivity [130].

In pregnant women, confirmation of active toxoplasmosis in reference laboratories can be achieved by the inoculation of body fluids or tissues in mouse or cell culture [18]. Mouse inoculation is absolutely sensitive; however, it requires the use of live animals, housing, euthanasia, autopsy and antibody testing. In addition, it takes a maximum of 6 weeks to obtain a diagnosis and is not widely available method for modem clinical laboratories [133, 134]. Cell culture is a practical method for the detection of *T*. *gondii* than mouse inoculation; however, it is relatively slow and may not be sensitive [135].

Unfortunately, in spite of the importance of cell culture and mouse inoculation for the detection of *T*. *gondii*, most researchers have not used these techniques in their researches. Moreover, polymerase chain reaction can be performed on amniotic fluid to detect *T*. *gondii*-specific DNA after 18 weeks of pregnancy [136].

### Limitations

The limitations of the present study include: 1) lack of large cohort of women with *T*. *gondii* infection, as compared to controls for the investigation of the association between abortion and *T*. *gondii* infection (because cross-sectional and case-control studies due to their nature, do not provide the possibility to explore the causal relationship), 2) the diversity of the diagnostic methods with different sensitivity and specificity, 3) The use of English articles due to lack of fluency in other languages.

### Conclusion

Since the pooled ORs for anti-*T*. *gondii* IgG and IgM antibodies in different studies among women who had spontaneous abortion were higher than controls, it shows a possible relationship between *T*. *gondii* and spontaneous abortion. Hence, emphasize on health education especially on the *Toxoplasma* transmission routes in the childhood, and performance of screening program using regular serologic tests during pregnancy could help physicians in the diagnosis, prevention, and treatment of toxoplasmosis and reduction of the economic burden of the disease on society.

## Supporting information

S1 Table PRISMA 2009 checklist. (DOC)

S2 Table NOS checklist. (DOCX)

S1 Fig The reported seroprevalence of anti- *T*. *gondii* IgM antibody in women who had a history of abortion. (TIF)

S2 Fig The reported seroprevalence of anti-*Toxoplasma* IgM antibody in women who had abortion in present pregnancy. (TIF)

S3 Fig Funnel plot to detect publication bias in cross-sectional studies showing IgG seropositivity rates of *T*. *gondii*. (TIF)

S4 Fig Funnel plot to detect publication bias in case-control studies showing IgG seropositivity rates of *T*. *gondii*. (TIF)

S5 Fig Funnel plot to detect publication bias in case-control studies showing IgG seropositivity rates of *T*. *gondii* after estimating censored studies. (TIF)

S6 Fig Sensitivity analysis for assessing the effect of each primary study on the total estimates in cross-sectional studies showing IgG seropositivity rates of *T*. *gondii*. (TIF)

S7 Fig Sensitivity analysis for assessing the effect of each primary study on the total estimates in case-control studies showing IgG seropositivity rates of *T*. *gondii*. (TIF)

S8 Fig Funnel plot to detect publication bias in cross-sectional studies showing IgM seropositivity rates of *T*. *gondii*. (TIF)

S9 Fig Funnel plot to detect publication bias in cross-sectional studies showing IgM seropositivity rates of *T*. *gondii* after estimating censored studies. (TIF)

S10 Fig Funnel plot to detect publication bias in case-control studies showing IgM seropositivity rates of *T*. *gondii*. (TIF)

S11 Fig Funnel plot to detect publication bias in case-control studies showing IgM seropositivity rates of *T*. *gondii* after estimating censored studies. (TIF)

S12 Fig Sensitivity analysis for assessing the effect of each primary study on the total estimates in cross-sectional studies showing IgM seropositivity rates of *T*. *gondii*. (TIF)

S13 Fig Sensitivity analysis for assessing the effect of each primary study on the total estimates in case-control studies showing IgM seropositivity rates of *T*. *gondii*. (TIF)
