# Supplementary material for: The global seroprevalence of anti-Toxoplasma gondii antibodies in women who had spontaneous abortion: A systematic review and meta-analysis
Source: PLoS Negl Trop Dis. 2020 Mar 13;14(3):e0008103. doi: 10.1371/journal.pntd.0008103 (PMC7069604; doi:10.1371/journal.pntd.0008103)
Supplement: S2 Table — (DOCX) [file pntd.0008103.s002.docx]

**Table S1. Quality assessment of included studies based on the Newcastle-Ottawa Scale.**

| No | Author | Yours | Type of study | Selection (3 points) | Comparability (2 points) | Outcome (3 points) | Total (scores) |
| --- | --- | --- | --- | --- | --- | --- | --- |
| 1 | Kimball AC et al | 1971 | Cross-sectional | 3 | 2 | 1 | 6 |
| 2 | Stray-Pedersen B and Lorentzen-Styr AM | 1977 | Case-control | 3 | 0 | 3 | 6 |
| 3 | Lolis D et al | 1978 | Case-control | 3 | 0 | 3 | 6 |
| 4 | Stray-Pedersen B et al | 1979 | Cross-sectional | 3 | 1 | 1 | 5 |
| 5 | Decavalas G et al | 1990 | Cross-sectional | 2 | 2 | 2 | 6 |
| 6 | Galván Ramírez ML et al | 1995 | Case-control | 2 | 0 | 3 | 5 |
| 7 | Sahwi SY et al | 1995 | Case-control | 3 | 0 | 2 | 5 |
| 8 | Djurkovic-Djakovic O | 1995 | Case-control | 3 | 1 | 3 | 7 |
| 9 | Al-Hamdani MM and Mahdi NK | 1996 | Case-control | 3 | 0 | 3 | 6 |
| 10 | Sanghi A et al | 1997 | Cross-sectional | 2 | 1 | 1 | 4 |
| 11 | Singh N et al | 1998 | Cross-sectional | 3 | 0 | 1 | 4 |
| 12 | Zargar AH et al | 1998 | Case-control | 3 | 0 | 3 | 6 |
| 13 | Qublan HS et al | 2002 | Cross-sectional | 3 | 2 | 2 | 7 |
| 14 | Elnahas A et al | 2003 | Cross-sectional | 3 | 2 | 2 | 7 |
| 15 | Nissapatorn V et al | 2003 | Cross-sectional | 3 | 2 | 2 | 7 |
| 16 | Chopra S et al | 2004 | Cross-sectional | 2 | 0 | 1 | 3 |
| 17 | Nimri L et al | 2004 | Case-control | 3 | 1 | 3 | 7 |
| 18 | Ertug S et al | 2005 | Cross-sectional | 3 | 2 | 2 | 7 |
| 19 | Surpam RB et al | 2006 | Case-control | 3 | 0 | 2 | 5 |
| 20 | Sebastian D et al | 2008 | Case-control | 3 | 0 | 2 | 5 |
| 21 | Al-Saeed MS et al | 2008 | Case-control | 1 | 0 | 3 | 4 |
| 22 | Barbosa IR et al | 2009 | Cross-sectional | 3 | 2 | 2 | 7 |
| 23 | Nijem KI and Al-Amleh S | 2009 | Cross-sectional | 2 | 2 | 2 | 6 |
| 24 | Dawood AL – Taie AA | 2009 | Case-control | 3 | 0 | 3 | 6 |
| 25 | Mohymen NA et al | 2009 | Case-control | 3 | 1 | 3 | 7 |
| 26 | Jaboory Hadi N | 2011 | Cross-sectional | 1 | 1 | 2 | 4 |
| 27 | Mousa DA et al | 2011 | Cross-sectional | 2 | 2 | 2 | 6 |
| 28 | Aziz FM and Drueish MJ | 2011 | Cross-sectional | 3 | 2 | 1 | 6 |
| 29 | Pavlinova J et al | 2011 | Cross-sectional | 1 | 1 | 1 | 3 |
| 30 | Jasim M et al | 2011 | Cross-sectional | 1 | 2 | 2 | 5 |
| 31 | Nissapatorn V et al | 2011 | Cross-sectional | 3 | 2 | 2 | 7 |
| 32 | Amin A et al | 2012 | Cross-sectional | 1 | 2 | 1 | 4 |
| 33 | Hajsoleimani F et al | 2012 | Cross-sectional | 3 | 2 | 2 | 7 |
| 34 | Malarvizhi A et al | 2012 | Cross-sectional | 3 | 2 | 2 | 7 |
| 35 | Elamin MH et al | 2012 | Case-control | 3 | 0 | 3 | 6 |
| 36 | Tammam AAE. et al | 2013 | Cross-sectional | 2 | 2 | 2 | 6 |
| 37 | Vado-Sol´ıs IA. et al | 2013 | Cross-sectional | 1 | 0 | 2 | 3 |
| 38 | Padmavathy M et al | 2013 | Cross-sectional | 1 | 1 | 1 | 3 |
| 39 | Ebrahimzadeh A et al | 2013 | Cross-sectional | 3 | 2 | 1 | 6 |
| 40 | De Moura FL et al | 2013 | Cross-sectional | 3 | 2 | 2 | 7 |
| 41 | Babaie J et al | 2013 | Cross-sectional | 3 | 2 | 2 | 7 |
| 42 | Chintapalli S and PadmajaI J | 2013 | Cross-sectional | 2 | 2 | 1 | 5 |
| 43 | Hussan BM | 2013 | Case-control | 3 | 0 | 3 | 6 |
| 44 | Abou-Gabal KM et al | 2013 | Case-control | 2 | 2 | 3 | 7 |
| 45 | Abedi M et al | 2014 | Cross-sectional | 2 | 2 | 1 | 5 |
| 46 | Alvarado-Esquivel et al | 2014 | Cross-sectional | 2 | 2 | 2 | 6 |
| 47 | Almushait MA | 2014 | Cross-sectional | 3 | 2 | 2 | 7 |
| 48 | Siddiqui N et al | 2014 | Case-control | 3 | 1 | 3 | 7 |
| 49 | Sultana M et al | 2014 | Case-control | 3 | 0 | 2 | 5 |
| 50 | Abbas HH et al | 2014 | Case-control | 2 | 0 | 3 | 5 |
| 51 | Awoke K et al | 2015 | Cross-sectional | 3 | 2 | 2 | 7 |
| 52 | Gelaye W et al | 2015 | Cross-sectional | 3 | 2 | 2 | 7 |
| 53 | Alvarado-Esquivel C et al | 2015 | Cross-sectional | 2 | 2 | 2 | 6 |
| 54 | Anubhuti et al | 2015 | Cross-sectional | 3 | 2 | 2 | 7 |
| 55 | Saki J et al | 2015 | Case-control | 3 | 0 | 3 | 6 |
| 56 | Ghasemi FS et al | 2015 | Case-control | 3 | 0 | 3 | 6 |
| 57 | Kamal AM et al | 2015 | Case-control | 3 | 1 | 3 | 7 |
| 58 | Hernández-Cortazar IB et al | 2016 | Cross-sectional | 1 | 2 | 2 | 5 |
| 59 | Mohamed K et al | 2016 | Cross-sectional | 3 | 2 | 2 | 7 |
| 60 | Mohaghegh MA et al | 2016 | Cross-sectional | 3 | 2 | 2 | 7 |
| 61 | Imam NFA | 2016 | Cross-sectional | 3 | 2 | 1 | 6 |
| 62 | Nazir MM et al | 2016 | Cross-sectional | 3 | 2 | 1 | 6 |
| 63 | Rasti S et al | 2016 | Case-control | 4 | 0 | 3 | 7 |
| 64 | Yasmeen A et al | 2017 | Cross-sectional | 3 | 2 | 2 | 7 |
| 65 | Negero J et al | 2017 | Cross-sectional | 3 | 2 | 2 | 7 |
| 66 | Matin S et al | 2017 | Cross-sectional | 2 | 2 | 1 | 5 |
| 67 | Abd El Aal AA. et al | 2018 | Cross-sectional | 2 | 1 | 1 | 4 |
| 68 | Costa GB et al | 2018 | Cross-sectional | 3 | 2 | 2 | 7 |
| 69 | Menati Rashno M et al | 2018 | Cross-sectional | 2 | 0 | 2 | 4 |
| 70 | Hafez Hassanain NA et al | 2018 | Cross-sectional | 3 | 2 | 2 | 7 |
| 71 | Çakmak BD et al | 2018 | Case-control | 3 | 0 | 3 | 6 |
| 72 | Kheirandish F et al | 2019 | Case-control | 3 | 0 | 3 | 6 |
