# Supplementary figures and images for: The global seroprevalence of anti-Toxoplasma gondii antibodies in women who had spontaneous abortion: A systematic review and meta-analysis
Source: PLoS Negl Trop Dis. 2020 Mar 13;14(3):e0008103. doi: 10.1371/journal.pntd.0008103 (PMC7069604; doi:10.1371/journal.pntd.0008103)

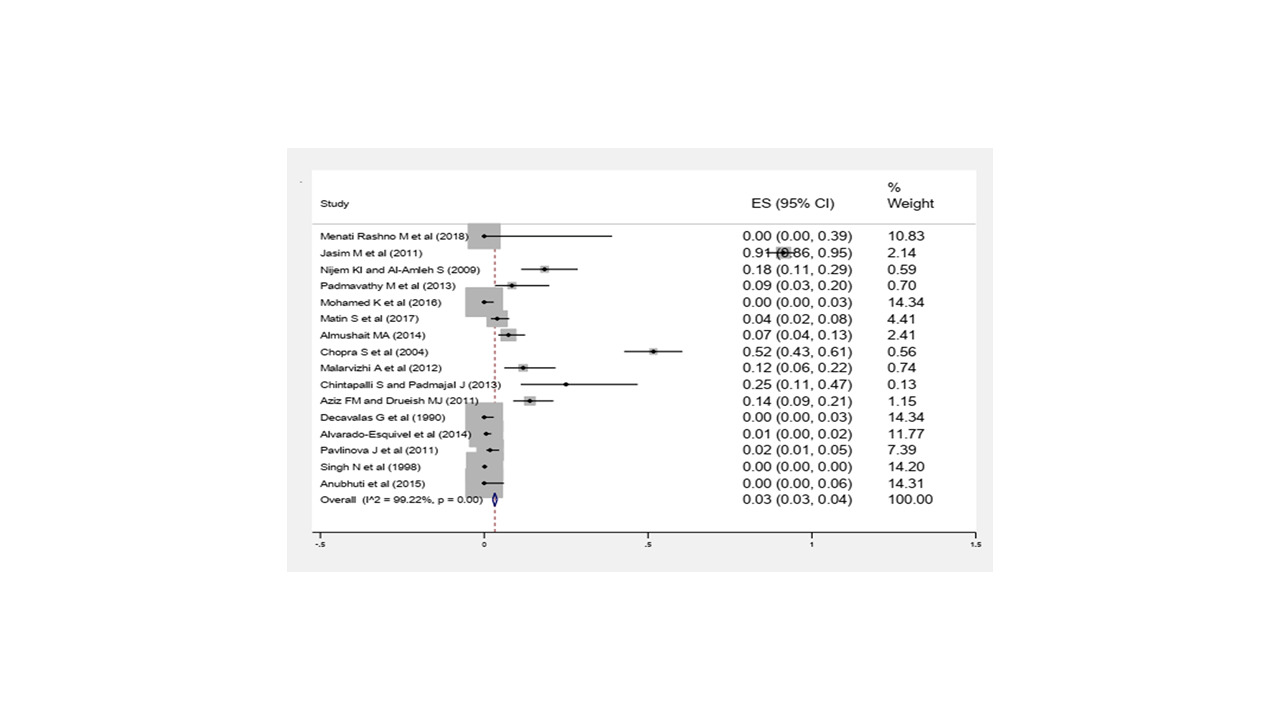

Supplement: S1 Fig — (TIF) [file pntd.0008103.s003.tif]

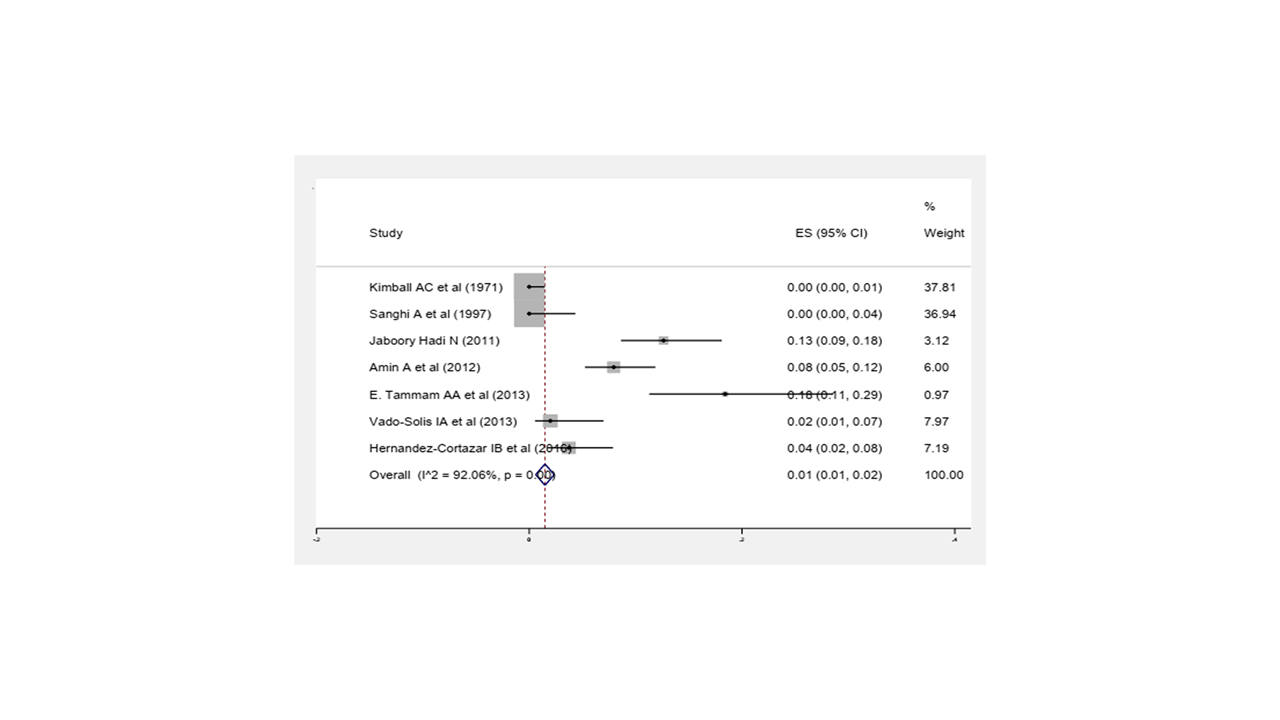

Supplement: S2 Fig — (TIF) [file pntd.0008103.s004.tif]

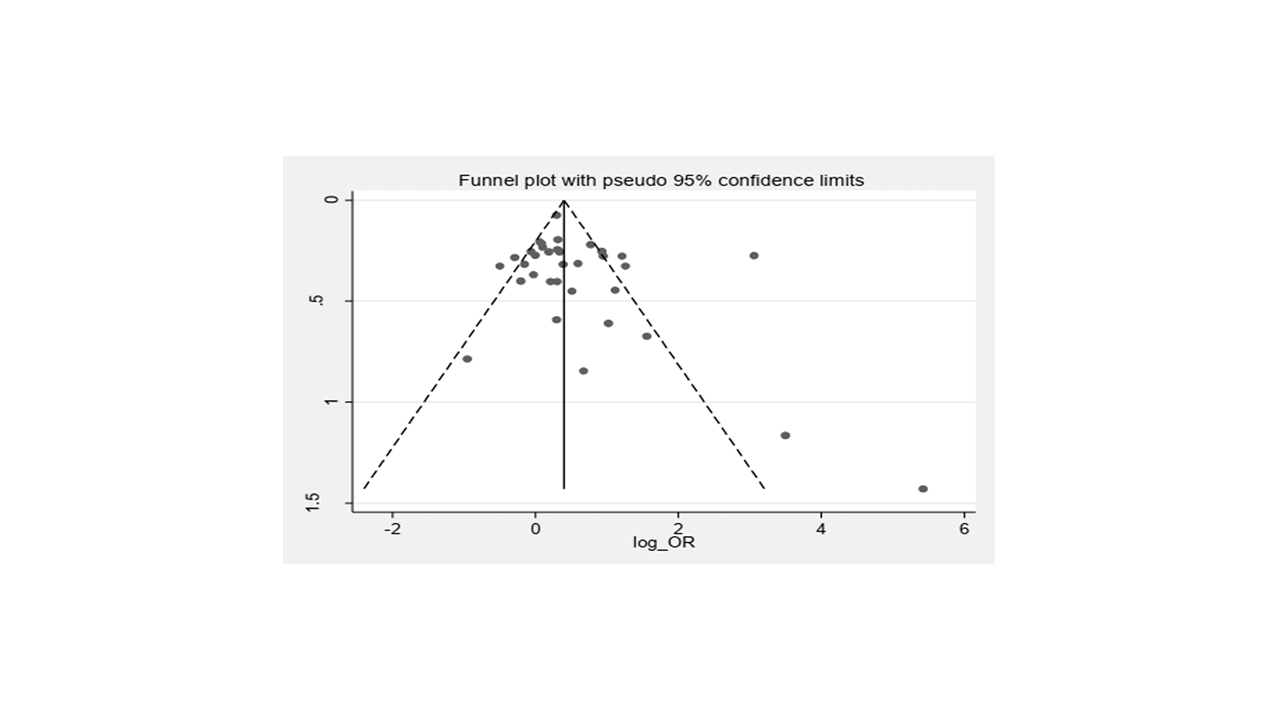

Supplement: S3 Fig — (TIF) [file pntd.0008103.s005.tif]

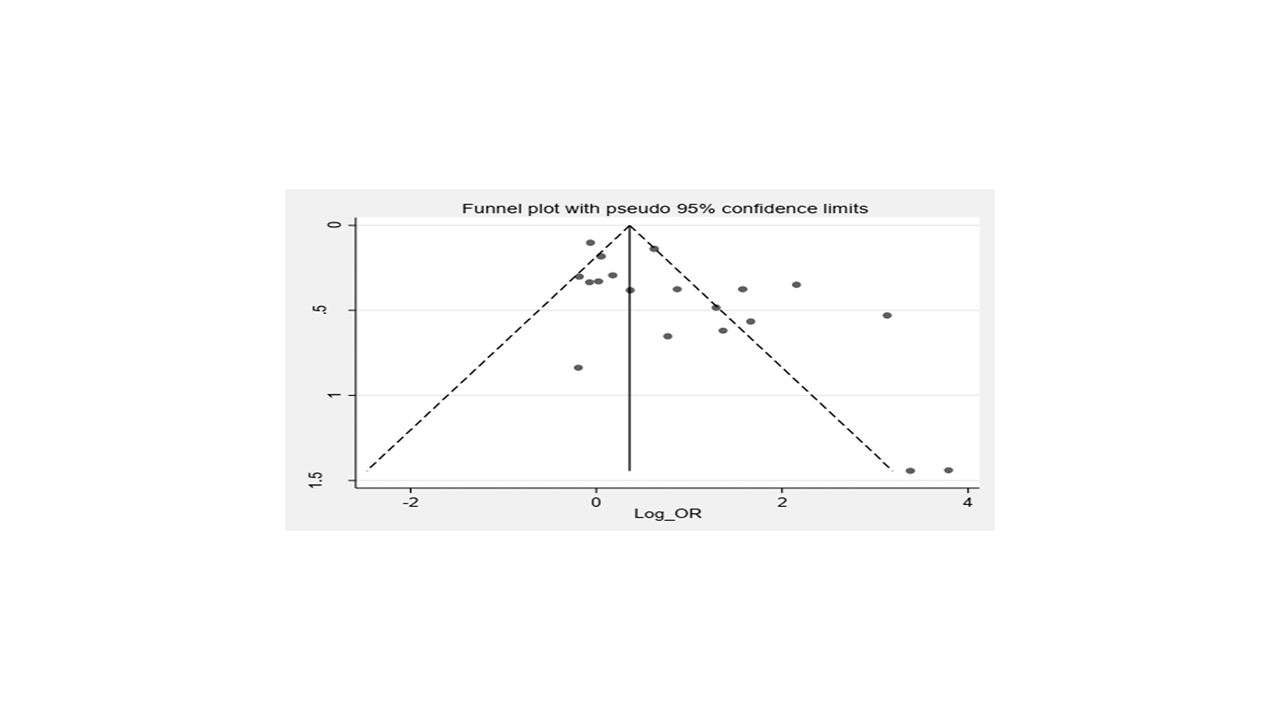

Supplement: S4 Fig — (TIF) [file pntd.0008103.s006.tif]

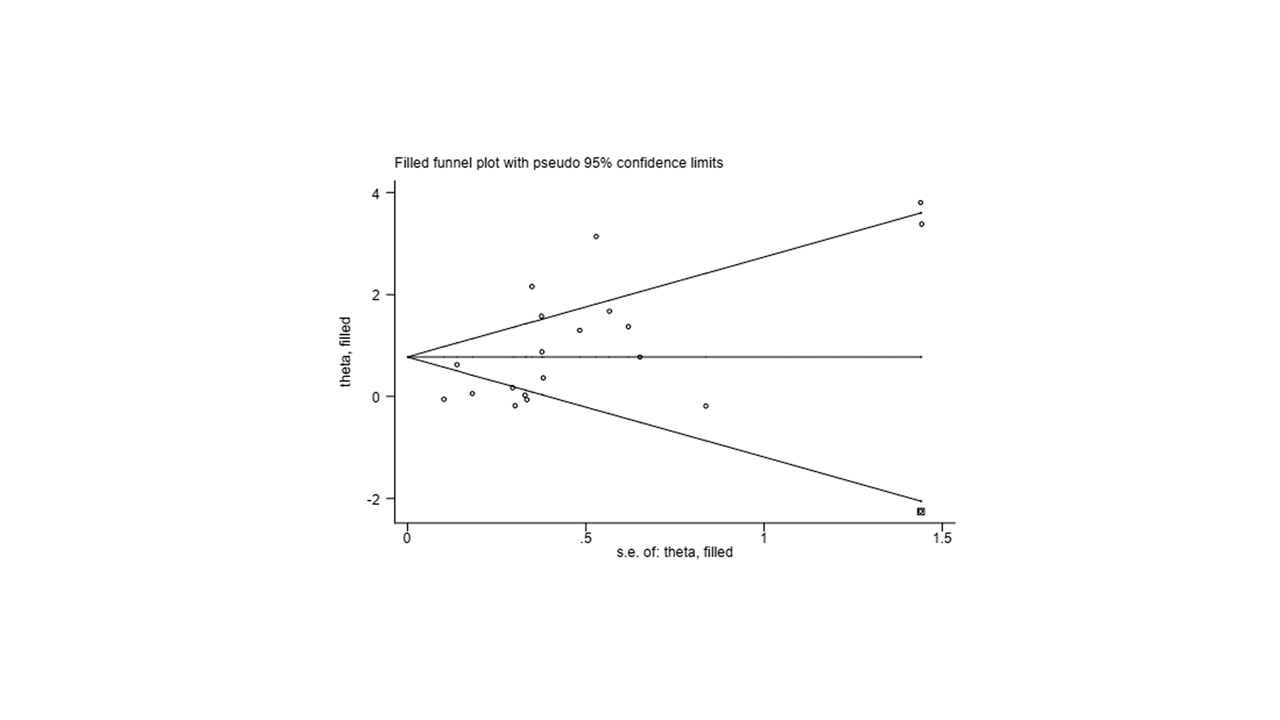

Supplement: S5 Fig — (TIF) [file pntd.0008103.s007.tif]

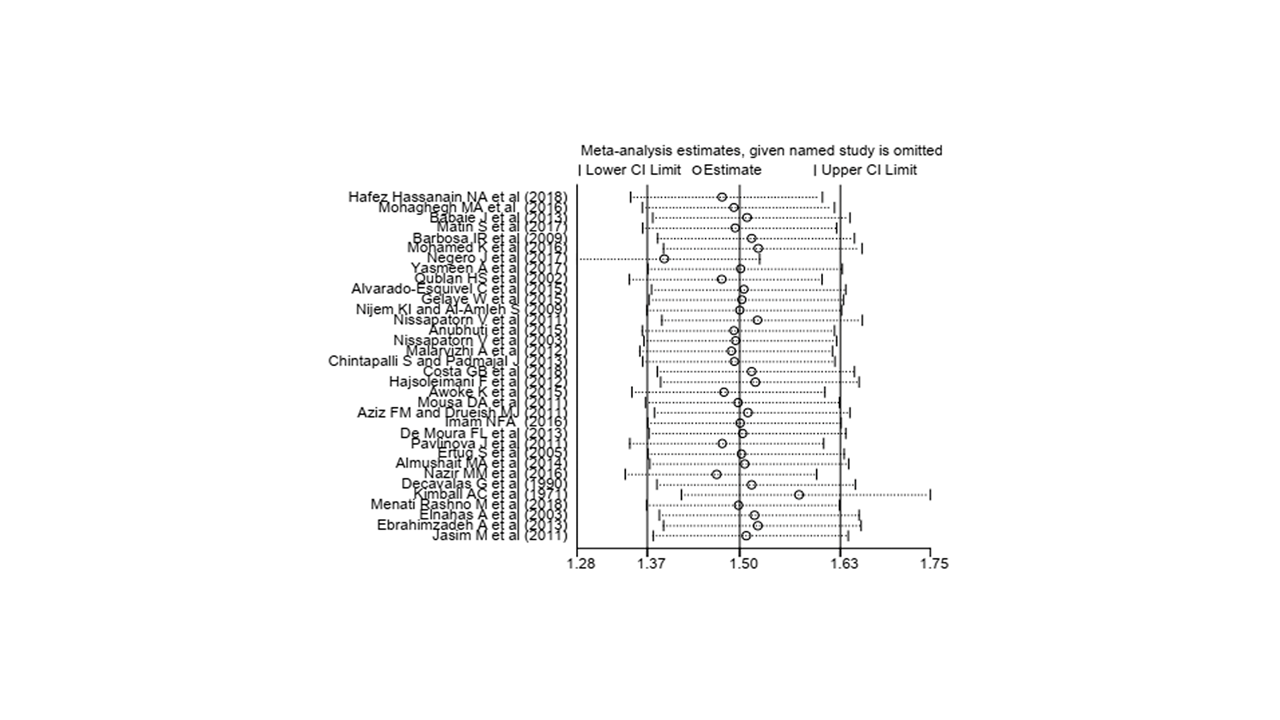

Supplement: S6 Fig — (TIF) [file pntd.0008103.s008.tif]

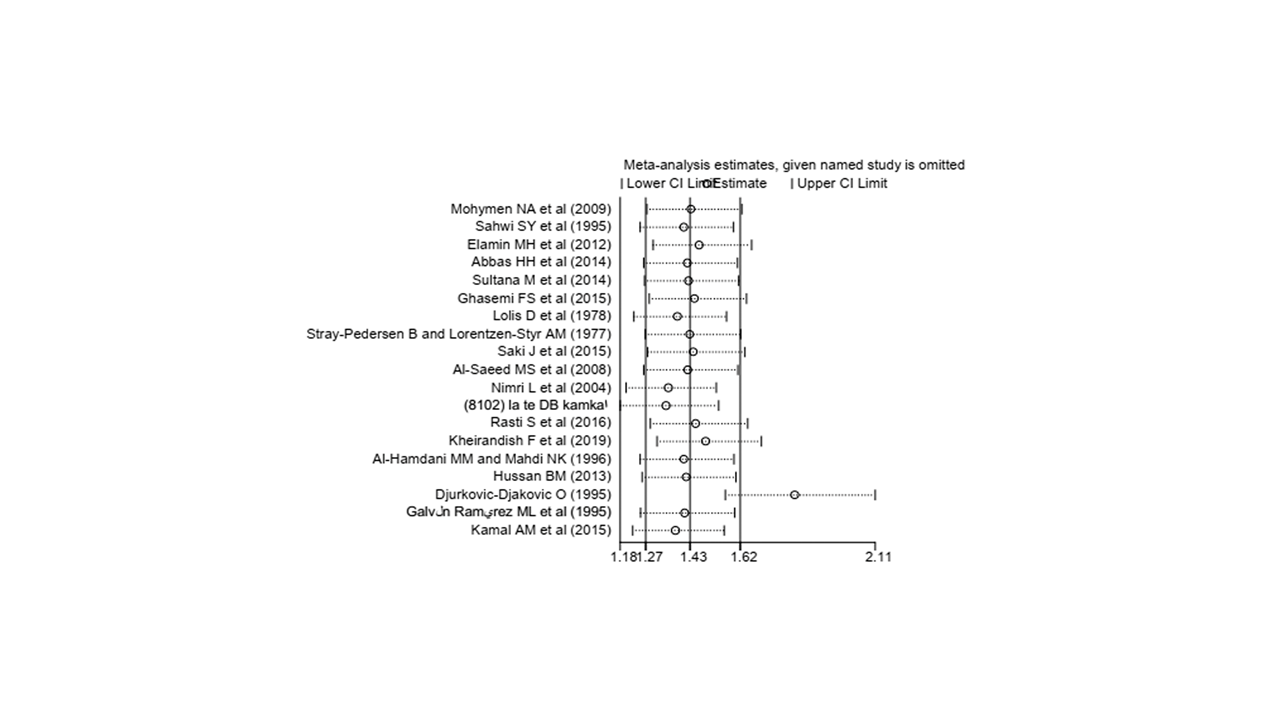

Supplement: S7 Fig — (TIF) [file pntd.0008103.s009.tif]

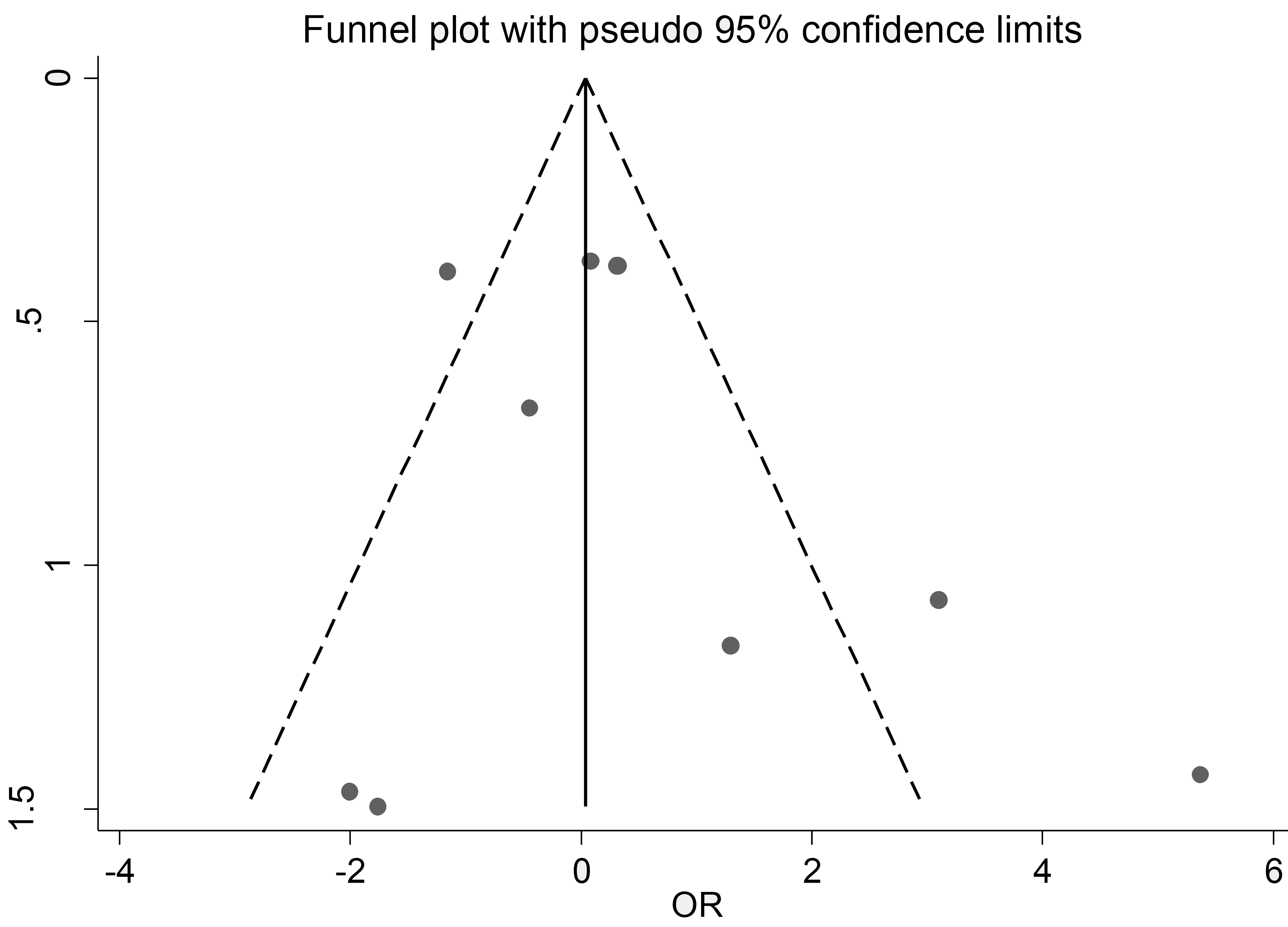

Supplement: S8 Fig — (TIF) [file pntd.0008103.s010.tif]

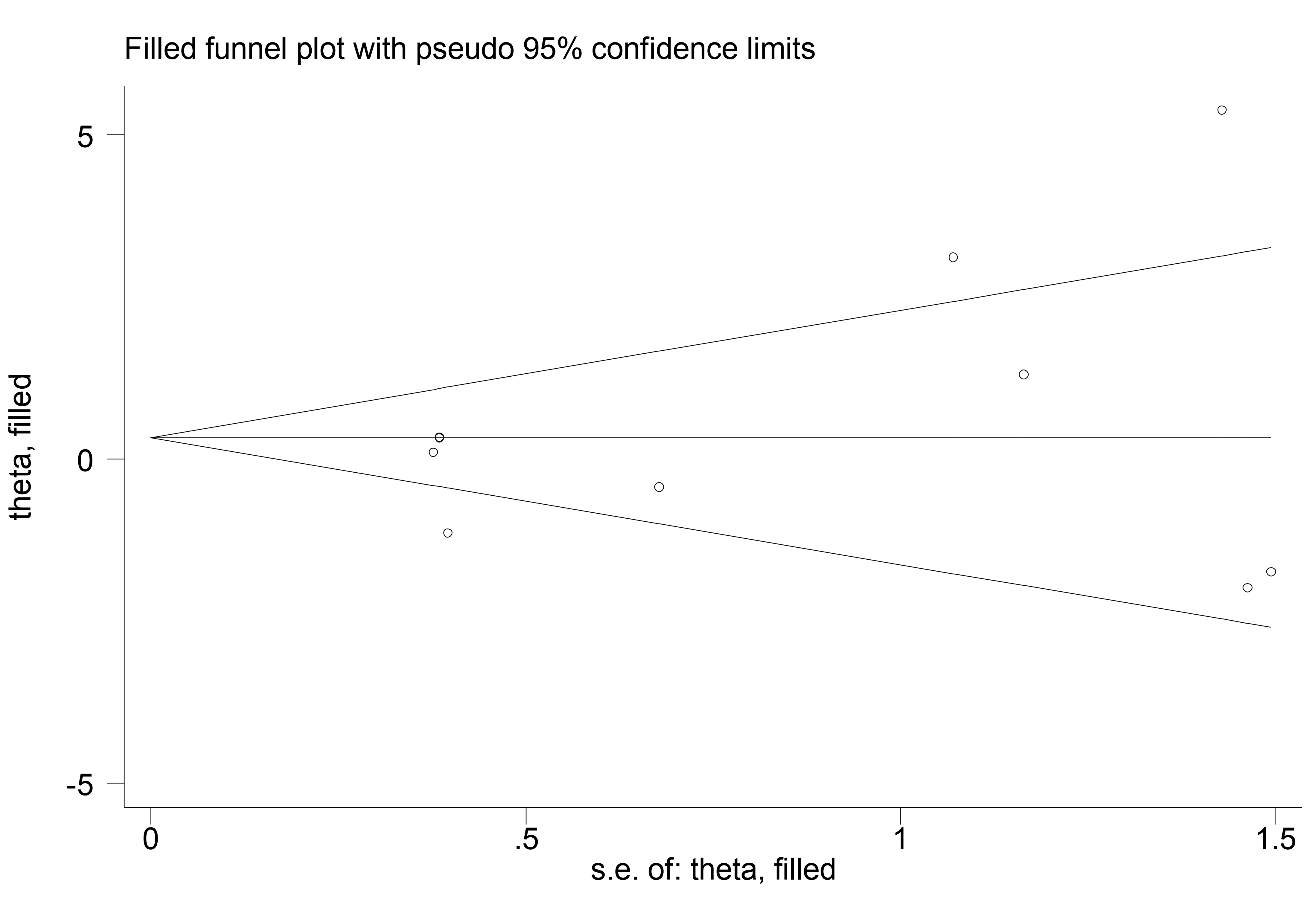

Supplement: S9 Fig — (TIF) [file pntd.0008103.s011.tif]

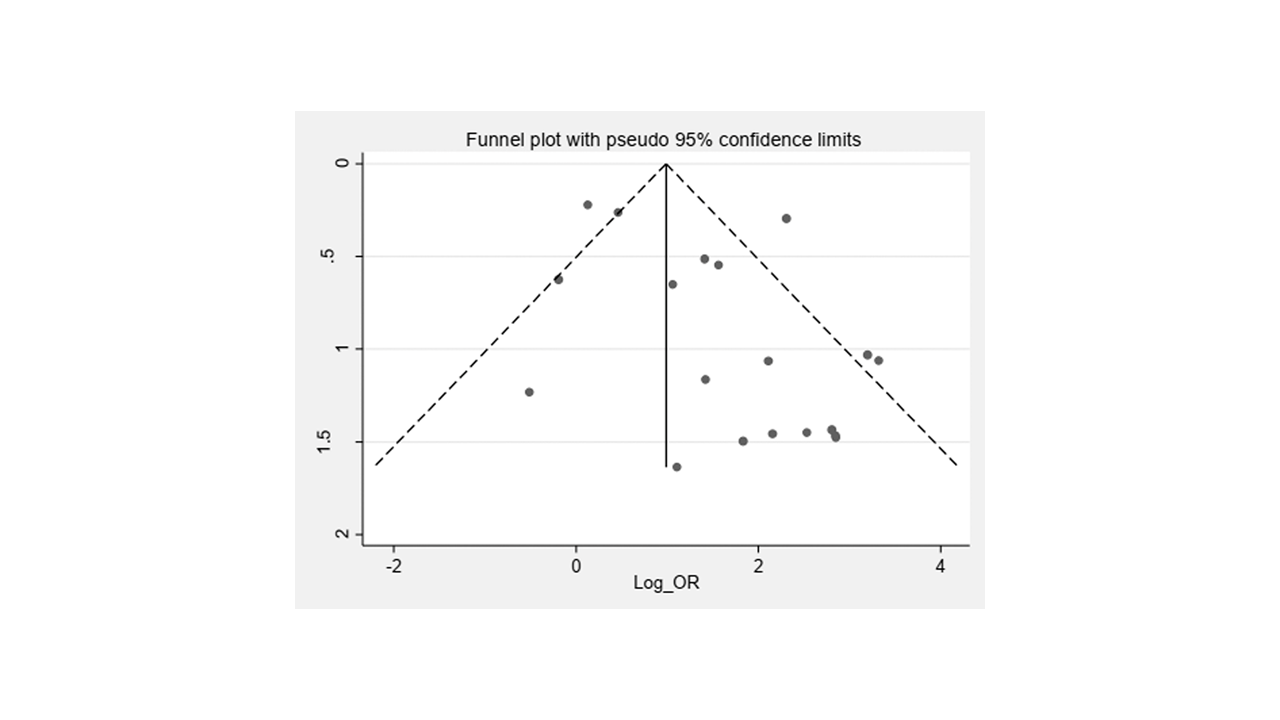

Supplement: S10 Fig — (TIF) [file pntd.0008103.s012.tif]

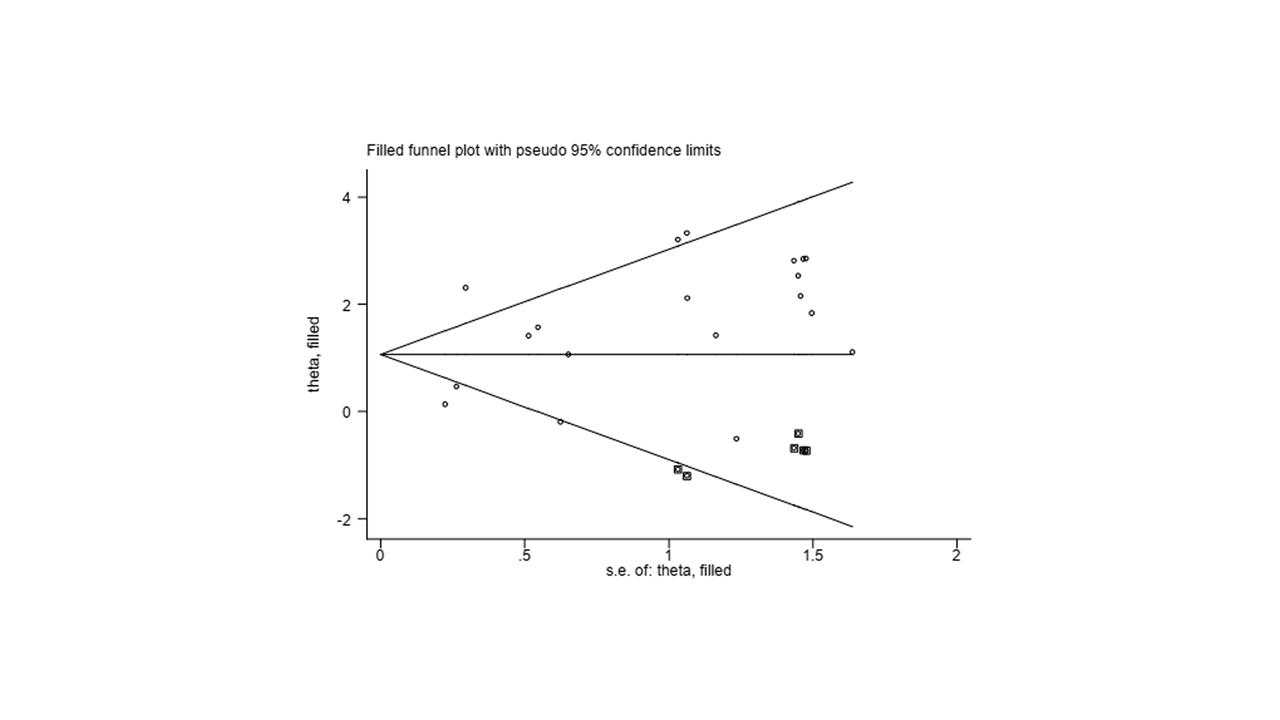

Supplement: S11 Fig — (TIF) [file pntd.0008103.s013.tif]

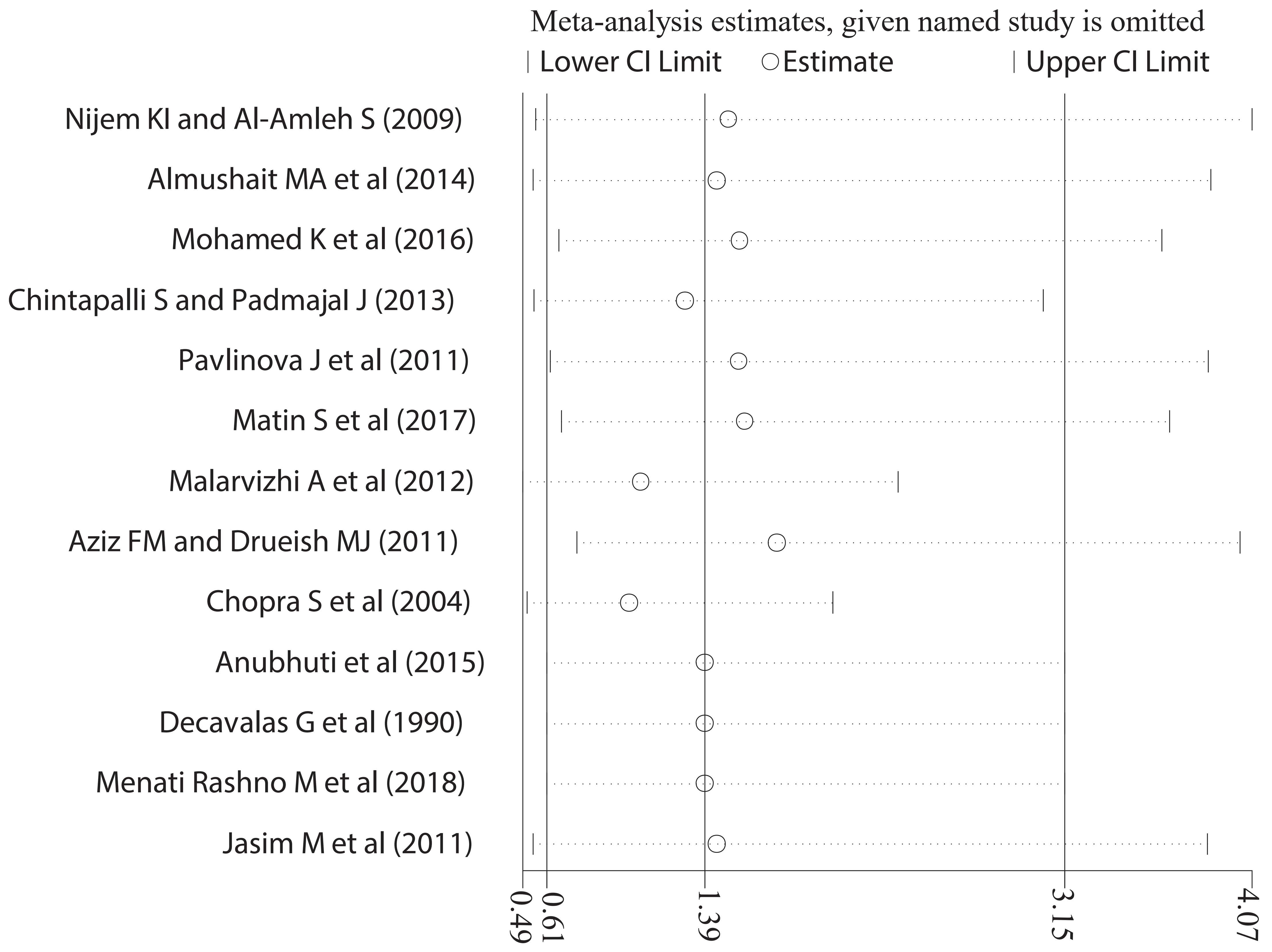

Supplement: S12 Fig — (TIF) [file pntd.0008103.s014.tif]

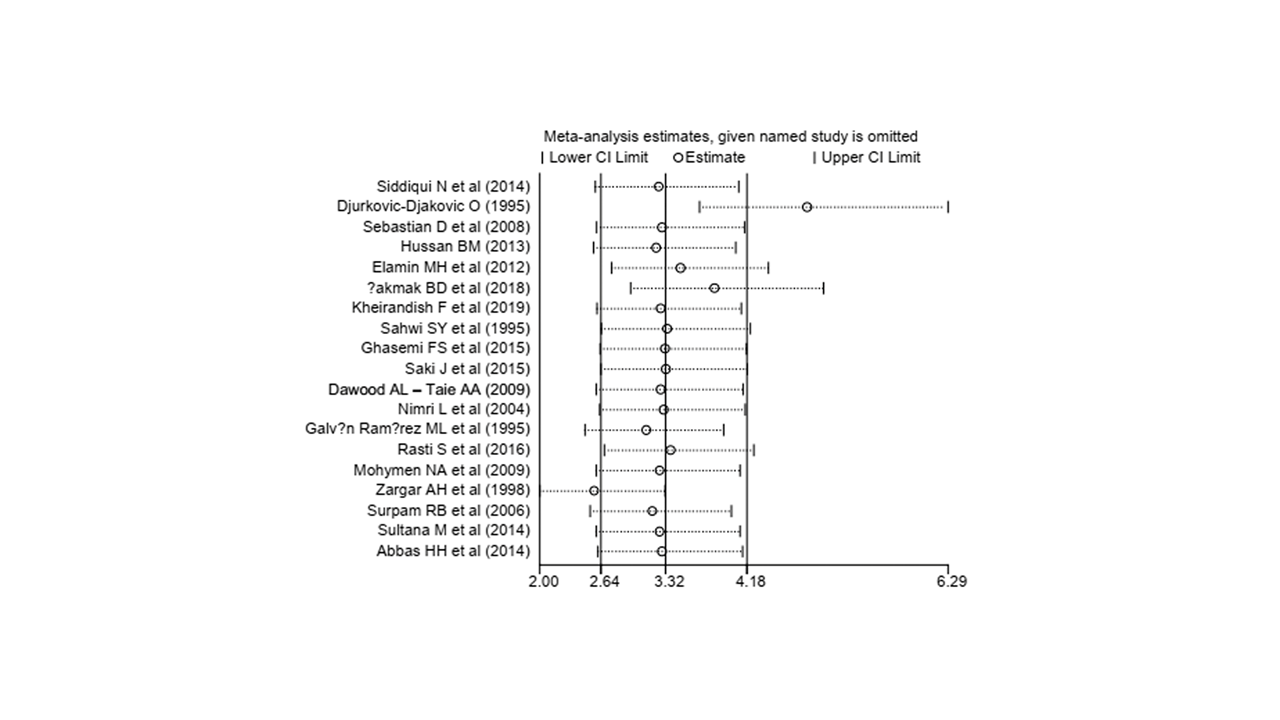

Supplement: S13 Fig — (TIF) [file pntd.0008103.s015.tif]
